# Development and Molecular Characterization of Low Phytate Basmati Rice Through Induced Mutagenesis, Hybridization, Backcross, and Marker Assisted Breeding

**DOI:** 10.3389/fpls.2019.01525

**Published:** 2019-11-26

**Authors:** Zia-ul- Qamar, Amjad Hameed, Muhammad Ashraf, Muhammad Rizwan, Muhammad Akhtar

**Affiliations:** ^1^Plant Breeding and Genetics Division, Nuclear Institute for Agriculture and Biology (NIAB), Faisalabad, Pakistan; ^2^Plant Breeding and Genetics Division, Nuclear Institute of Agriculture (NIA), Tando Jam (Sindh), Pakistan; ^3^Soil and Environmental Sciences Division, Nuclear Institute for Agriculture and Biology (NIAB), Faisalabad, Pakistan

**Keywords:** gamma rays, genetic biofortification, mineral deficiency, mutant alleles, *Oryza sativa*, phytic acid

## Abstract

Breeding low phytate crops is the most viable solution to tackle mineral deficiencies. The objective of the present study was to develop high yielding, low phytate (lpa) basmati rice cultivars. Three homozygous lpa mutants, Lpa5, Lpa9, and Lpa59, were developed through induced mutations (gamma rays ^60^Co) and identified by colorimetric and High Performance Liquid Chromatography (HPLC) analysis. These mutants showed 54%–63% reduction in phytic acid but had poor germination and yield. To improve these traits, hybridization and back cross breeding involving Lpa5, Lpa59, and parent cultivar Super Basmati were performed and F_2:3_, F_3:4_, BC_1_F_2:3_, and BC_1_F_3:4_ generations were developed and screened to target the objective. Within the F_2:3_, homozygous (226), heterozygous (65), and wild type (46) lpa recombinants were identified. Within the homozygous lpa category, four recombinants (Lpa5, Lpa6, Lpa7, and Lpa30) showed improved germination. Within the F_3:4_ generation, 86 homozygous lpa recombinants were identified. Further selection, on the basis of better plant type and the low phytate trait resulted in the selection of 38 recombinants. Grain quality and cooking characteristics of these selected recombinants were comparable as compared to parent cultivar. Within the BC_1_F_2:3_ generations, two homozygous Lpa recombinant lines, Lpa141, and Lpa205, were selected out of 220. Screening of the BC_1_F_3:4_ generation for the desirable agronomic and low phytate trait also resulted in the selection of two homozygous lines. Finally, seven recombinants i.e. Lpa12-3, Lpa111-1, Lpa141, Lpa56-3, Lpa53-4, Lpa99-2, and Lpa205-4 out of 42 homozygous low phytate lines were selected on the basis of yield improvement (4%–18%) as compared to parent cultivar. Association analysis suggested that further selection based on primary branches per plant, panicle length and productive tillers per plant would further improve the paddy yield. For molecular characterization of the Lpa trait, previously reported Lpa1-CAPS and Lpa1-InDel and functional molecular markers were applied. Results indicated the absence of the Z9B-Lpa allele and XS-Lpa mutation in the OsMRP5 gene in tested mutants, possibly suggesting that there may be new mutations or novel alleles in tested mutants that need to be identified and then fine mapped for subsequent utilization. To our knowledge, this is the first report of low phytic acid rice mutant development and their improved germination and yield through backcross breeding in basmati rice.

## Introduction

Phytic acid, myo-inositol 1, 2, 3, 4, 5, 6 hexakisphosphate (IP 6) is the major storage compound of phosphorous (P) in plants. It is present in all cereals and legume grains. The low phytic acid (LPA) trait addresses an urgent goal for genetic improvement of rice because of human physiological disorders like anemia, osteoporosis, and premature abortion especially in countries which consume rice as the staple diet. The phenomenon is also termed as hidden hunger or mineral malnutrition. Basically, phytic acid is a strong chelating agent and binds important minerals such as calcium, magnesium, iron, and zinc. When a mineral binds to phytic acid, it becomes insoluble, precipitates, and is not absorbed in the intestines of non-ruminants, including humans. This process can therefore contribute to mineral deficiencies in people whose diets rely on these foods for their mineral intake, such as those in developing countries or could even prevent gut absorption when high mineral content foods are eaten at the same time as high phytate rice. In cereals, phytic acid bound phosphorus typically represents about 75% and inorganic acid P about 5% of seed total P (Lott et al., 1995). In LPA mutants, isolated in the last few years in maize, barley, and rice, such ratios are altered (Raboy, 2002; Dorsch et al., 2003; Raboy, 2007). Mutant seeds have normal levels of total P, but greatly reduced levels of phytic acid P (Raboy et al., 2001). Low phytate crops have the potential to alleviate environmental and nutritional problems associated with phytic acid in human and animal feeds (Ertl et al., 1998). In Pakistan, rice is the second staple food crop after wheat and covers more than 2.810 million hectares of cropped area with an average yield of 2562 kg/hectare (Anonymous, 2018–19). With total production of 7.202 million tons, rice accounts for 3% of the total value added in agriculture and 0.6% of G.D.P. in Pakistan (Anonymous, 2018–19). Bio-fortified intervention through rice breeding can help to improve the nutritional status of the poor of the nation on a sustainable basis. Induced mutagenesis (physical/chemical) can be used to generate LPA mutants in rice (Raboy, 2009). A number of rice LPA mutant lines with ∼30% to ∼63% reduction in phytic acid have been developed with the aim of increasing the bioavailability of essential micronutrients (Rutger et al., 2004; Liu et al., 2007; Zhao et al., 2008a). Different causative LPA mutations have also been identified in previous studies, e.g., one mutation (XS-*lpa*) of myo-inositol kinase (Kim et al., 2008), two allelic mutations (KBNT-*lpa* and XQZ-*lpa*) of *LOC_Os02g57400* gene (Zhao et al., 2008b). Xu et al. (2009) also reported two allelic mutations (Z9B-*lpa* and MH-*lpa*) of the *OsMRP5* gene. Jiang et al. (2019) observed significantly lower levels (-10.1% and -32.1%) of phytic acid in two rice mutant lines ositpk6_1 and _2 as compared with the wild type.

Primary LPA mutants often feature inferior agronomic performance in terms of field emergence and grain yield (Zhao et al., 2008a; Raboy, 2009). Different studies have demonstrated that mutations in phytic acid genes impairs plant growth, lower grain yield, and reduce seed viability compared with wild type (Rutger et al., 2004; Jiang et al., 2019; Zhou et al., 2019). It has also been reported that germination and grain yield can be improved through breeding and selection (Spear and Fehr, 2007); however, extensive efforts are needed in breeding LPA rice cultivars for commercial production. Therefore, the primary objective of the current project was to develop, characterize, and improve the germination and grain yield of LPA mutants by hybridization, back cross and marker assisted breeding, and subsequent selection of low phytate recombinants with improved traits of interest. During the research project, we attempted to develop low phytate basmati rice with promising yield and germination.

## Materials and Methods

### Study Site and Experimental Material

The research work was conducted at the Plant Breeding and Genetics Division, Nuclear Institute for Agriculture and Biology (NIAB), Faisalabad, Pakistan between the years 2009 and 2017. The experimental material consisted of three low phytate mutant lines (Lpa5, Lpa9, and Lpa59) with poor germination and yield and non-low phytate Super Basmati with normal germination and yield. These low phytate mutants (∼54-63% reduction in phytic acid) were developed using gamma radiations (^60^Co source) at 250 Gy dose under an IAEA project RAS7/014. The breeding history of the low phytate basmati rice mutants with improved germination and yield is presented in **Table 1** and details are as follows.

**Table 1:** Breeding history of the low phytate basmati rice mutants with improved germination and yield.

Year	Generations	Breeding task along with selection criteria	Total population/mutants/Crosses/Plants/Progenies	Selections
2009–10	Low phytate mutant lines in Super Basmati background	Low phytate mutants (∼54-63% reduction in phytic acid) with poor germination and yield were obtained through gamma radiations (^60^Co source) at 250 Gy dose under an IAEA project RAS/7/014	80000	03 (Lpa5, Lpa 9 and Lpa59)
2010–11	F_0_	Two Lpa mutant lines (Lpa5 and Lpa59) were selected for crossing with Super Basmati due to their relatively better germination and yield. Following crosses were attempted: Lpa5 × Super Basmati, Lpa59 × Super Basmati, Super Basmati × Lpa5, Super Basmati × Lpa59	20	Seed bulked crosswise
2011–12	F_1_	Raising and selfing of F_1_ generation in field	72	Seed bulked crosswise
2012–13	F_2:3_	Single plant selection on the basis of low phytate trait and germination percentage	337 (Homozygous low phytate 226, heterozygous 65 and wild type rejected 46 plants)	291
2013–14	F_3:4_	Selection of single plant progenies on the basis of low phytate trait	291 (Homozygous progenies 86, heterozygous 71and wild type rejected progenies 134)	86
		Selection of single plant progenies on the basis of better plant type, grain quality and presence of low phytate trait	86	38
2012–13	BC_1_ F_1:2_	Four back cross populations were obtained by crossing of parent variety super basmati with F_1_ generation of all four cross combinations.	56	Seed bulked crosswise
2013–14	BC_1_ F_2:3_	Raised in the field and single plants were selected on the basis of yield and yield contributing traits	220	1^st^ BC_1_ F_2:3_ (09) 2^nd^ BC_1_ F_2:3_ (38) 3^rd^ BC_1_ F_2:3_ (11) 4^th^ BC_1_ F_2:3_ (53)
		Screening for low phytate trait and selection of homozygous plants	111	02
2014–15	BC_1_ F_3:4_	Out of 111 plant progenies from four BC_1_ F_2:3_ populations, 109 were raised in the field. Selections were continued for desirable agronomic and homozygosity for the low phytate trait.	109	02
2015–16		Evaluation of selected homozygous low phytate lines in preliminary yield trials and selection of best lines on the basis of yield and associated traits	42	07
2016–17		Identification and characterization of Lpa mutations using molecular markers	–	–

### Crossing of Super Basmati and Low Phytate Mutants

Parental genotypes (Lpa5, Lpa59, and Super Basmati) were sown in the nursery at an interval of 1 week to ensure the availability of the pollen for crossing. Low phytate mutant Lpa9 was not included further in the crossing program due to its very low germination and yield as compared to Lpa5 and Lpa59. About 25 spikelets were kept on the panicle and the rest were removed. Each spikelet was cut with scissors (1/3rd of the top portion) and anthers were removed. Spikelets were covered with a butter paper bag and next day, spikelets were dusted with pollen of the low phytate parent. In the crossing program, low phytate mutants were used as the donor parent and Super Basmati was used as the recipient parent.

### Raising/Screening of Different Populations in the Field

F_1_ generation was developed by crossing the non-low phytate Super Basmati plant with selected low phytate mutant plants of Super Basmati. Reciprocal crosses (Lpa5 × Super Basmati, Super Basmati × Lpa5; Lpa59 × Super Basmati, Super Basmati × Lpa59) were attempted to eliminate potential maternal effects in the succeeding generations. Each F_1_ plant was selfed to develop the F_2:3_ generations. Plant to row progenies of F_2:3_ generations were established to develop the F_3:4_ generations. Each segregating generation was screened by a high throughput colorimetric assay technique for the low phytate trait.

Four BC_1_F_2:3_ populations, (Lpa5 × Super Basmati) × Super Basmati; (Super Basmati × Lpa5) × Super Basmati; (Lpa59 × Super basmati) × Super Basmati; (Super Basmati × Lpa59) × Super Basmati were developed by back crossing the direct and reciprocal F_1_ populations of low phytate and Super Basmati parents. All four populations were raised in the field and regular management operations were conducted as and when required. At maturity, single plants, in variable number from each population, were selected on the basis of desirable plant attributes. Colorimetric assays were performed to detect the homozygous low phytate recombinants.

### Germination Tests of Different Segregating Generations and Selected Low Phytate Recombinants

About 200 seeds, of each F_2:3_ plant were sown under the field conditions in the nursery area. Seeds were covered with a thin layer of farm yard manure. Wheat straw was spread over the farm yard manure to save the seed from birds and direct contact with sprinkled water. Sprinkling of water was continued for 3 days. After 3 days, seedlings emerged and were counted to estimate the germination percentage.

$$\mathrm{Germination}\;\left(\%\right)=\frac{\mathrm{Number}\;of\;\mathrm{seeds}\;\;\mathrm{that}\;\mathrm{germinated}}{\mathrm{Number}\;of\;\mathrm{seeds}\;on\;\mathrm{the}\;\mathrm{tray}}\;\times \;100$$

Germination tests of 38 low phytate progenies/lines (homozygous for the low phytate trait) selected from F_3:4_ generation, were conducted under controlled conditions. Fifty seeds of each progeny having 12%–14% moisture were sown in plastic pots of 13 × 5 cm size and filled with autoclaved sand. Pots were kept at 25 ± 5°C and 12 h day light. Seeds were covered with a 2 cm layer of sand to mimic the underground soil conditions. Throughout the experiment, seeds were kept moist by applying a gentle stream of water daily in the morning by using a sprinkler. For up to 10 days, pots were checked to keep the sand moist and count the number of seeds germinated. By the end of 10 days, germinated seeds were counted and the germination percentage was computed. No germination tests were performed for the backcross populations. These populations were screened for desirable agronomic traits and homozygosity for the low phytate trait.

### Estimation of Grain Quality Parameters

Quality parameters of 38 low phytate lines selected from the F_3:4_ generation along with parent cultivar Super Basmati were also assessed. Physical grain parameters (Paddy length, width, grain length, width) were measured on a photographic enlarger as described by Shamim et al. (2017). The grain elongation ratio was calculated by dividing the average length of cooked grain by the average length of uncooked grain. For milling recovery parameters, head rice recovery (%) was determined as percentage of whole milled grains with respect to brown rice (Akhter et al., 2015). For cooking parameters, cooked grain length (mm) and bursting percentage were measured as described by Akhter et al. (2015). Aroma of the selected progenies and cultivar Super Basmati was tested in the leaf and grain as described by Sun et al. (2008). Aroma in leaves was determined at the tillering stage by excising 2 g of two or three leaves from each individual plant then cutting the tissues into pieces and placing them in petri dishes containing 10 ml of 1.7% potassium hydroxide (KOH). The petri dishes were kept at room temperature for about 10 min and then opened one by one, smelled and rated for the presence or absence of aroma. Aroma in grain was assessed by chewing and tasting of rice grain by a rating panel which consisted of four to five people selected for their ability to differentiate between aroma and non-aroma.

### Field Trial of Selected Low Phytate Recombinants

Yield performance of stable low phytate lines with better yield and germination was tested in a randomized complete block design with three replications and 20 cm plant to row spacing. Cultivar Super Basmati was included as a check. Thirty-day-old seedlings were transplanted and standard plant protection and agronomic measures were performed throughout the growing season. At maturity three plants per replication were harvested. Data were recorded on yield and various yield related traits.

### Colorimetric Assay to Detect the Low Phytate Recombinants

A simple screening procedure for detecting putative low phytate rice mutants was used as described by Raboy et al. (2001). Briefly, eight seeds per plant were ground, individually using mortar and pestle. The resulting flour was extracted overnight in 200 µl of 0.4 M HCl per mg (approximate seed wt.) at 4 °C. Samples (10 µl) of these extracts were mixed with 90 µl of distilled water and 100 µl of colorimetric reagent as described by Chen et al. (1956). Assays were incubated at room temperature for approximately 1 h so that individual seed extracts could be visually observed and scored for the assessment of high inorganic phosphorus (HIP). The assays were allowed to develop for 1 h at ambient (room) temperature. Seed extracts showing higher strength of blue color than the 3^rd^ P-standard (465 ng P) were identified as potential carrier of low phytate mutation/gene.

### Determination of Seed Phosphorus Levels

The total phosphorus in the seeds was extracted using the alkaline peroxodisulfate digestion method (Woo and Maher, 1995). An equal number of individual seed samples were crushed and 2 ml of digestion reagent (0.27 M potassium peroxodisulfate/0.24 M sodium hydroxide) and 10 ml of deionized water were added. The sample mixture was autoclaved at 120 °C for 60 min. A 1 ml aliquot of the extract of each sample was centrifuged at 20,000 g for 10 min, followed by spectrophotometric assay at 800 nm (Chen et al., 1956). For the analysis of the inorganic phosphate (Pi) levels, individual seed sample were ground to powder. The crushed powder was further extracted with 12.5% (w/v) trichloroacetic acid containing 25 mM MgCl_2_ and centrifuged at 20,000 g for 10 min. The supernatant was collected, and the Pi level was determined using 4 ml of freshly prepared Chen’s reagent (6 N H_2_SO_4_, 2.5% ammonium molybdate, and 10% ascorbic acid). The absorbance of the resulting colored complex was measured at 800 nm (Chen et al., 1956).

### Phytic Acid Content Analysis by HPLC

HPLC analysis of phytic acid was based on metal replacement reaction of the phytic acid from colored complex of iron (III)–thiocyanate and the decrease in concentration of the colored complex was monitored (Dost and Tokul, 2006). Prior to extraction, each sample was homogenized well using a mortar and pestle. 200 mg of each sample was weighed and extracted with 0.5 M HCl by continuous stirring for 1 h at room temperature followed by centrifugation at 4000 rpm for 15 min. The supernatant was collected and stored at 4 °C for further use. For HPLC analysis, 0.1 ml of the sample extract was placed in a 3 ml glass tube and mixed with 0.9 ml ultra-pure water and 2 ml of iron (III)–thiocyanate complex solution. The mixture was stirred in a 40 °C water bath for 2.5 h and cooled at room temperature. After centrifuging the mixture for 5 min, 20 ml of the supernatant was injected onto the column of a reverse-phase HPLC system (Waters, USA). The mobile phase was a mixture of 30% acetonitrile in water including 0.1 M HNO_3_, and the flow was adjusted to 1 ml for 21 min. The peak of iron (III)–thiocyanate was detected at 460 nm. The phytic acid concentration was calculated using the calibration curve prepared with a phytic acid standard (Sigma Aldrich; P0109).

### Molecular Characterization of Lpa Mutations

Genomic DNA was extracted from the M_8_ generation of stable low phytate mutants and from recombinant low phytate lines homozygous for the Lpa trait in the F_2:3_ generation. DNA was isolated from 200 mg sample of young leaf tissue harvested from seedlings by using the method described by Hameed et al. (2004). Concentration and purity of extracted DNA were checked through Spectrophotometer (U-2800 Spectrophotometer) at an absorbance of 260 nm and 280 nm. Using the extracted DNA, dilutions were prepared for subsequent PCR analysis. The PCR reactions were performed for Simple Sequence Repeats (SSR), InDel and CAPS analysis.

### SSR Analysis

PCR reactions were performed in a 20 µl reaction volume containing 2 µl of 10x PCR buffer, 2 µl of 2 mM dNTPs, 1.2 µl of 25 mM MgCl_2_, 0.20 µl of 5 unit/µl of the enzyme Taq polymerase, 0.4 µl of 20 µM each of the two primers, and 4 µl of 25 ng/µl template DNA. Amplifications were performed in a BIORAD T-100 gradient thermal cycler (Bio-Rad Laboratories, USA). The amplification cycles consisted of 40 cycles with denaturation at 94°C for 1 min, annealing at 50°C to 60°C (depending upon the primer pair) for 1 min and extension at 72°C for 1 min. The final extension was done at 72°C for 7 min. Amplification products were separated on non-denaturing 6% polyacrylamide gels and fragments were detected by ethidium bromide staining. Gels were documented using a UVI pro Platinum 1.1 System and UVIB and Map software (UVItec Ltd., Cambridge, UK).

### InDel and CAPS Analysis

PCR reactions were performed in a 25 µl reaction volume containing the following components: 100 ng of template DNA, 200 µM of each of the four dNTPs, 1x Taq polymerase buffer, 1 unit Taq polymerase, 2 mM MgCl_2_, and 0.25 µM each of the two primers. Amplifications were performed in a BIORAD T-100 gradient thermal cycler (Bio-Rad Laboratories, USA).

With respect to the CAPS markers for XS-Lpa mutation, the primers P3F: CTTGTGGCTACATTTGGTTT and P3R: AGGATGAGGGACGGCTA were used (Tan et al., 2013).The amplification cycles consisted of 32 cycles with denaturation at 94°C for 1 min, annealing at 52°C for 1 min and extension at 72°C for 90 s. The final extension was done at 72°C for 10 min. Amplification products were digested with *Hae*III and then separated on 1% agarose gels and fragments were detected by ethidium bromide staining. Regarding InDel markers, for ZB-Lpa mutation, the primers P5F: GGTGCCCAGCTACTCCTTCTC and P5R: GCGAAGATTATATCATTGCCTG were used as described by Tan et al. (2013). The amplification cycles consisted of 35 cycles with denaturation at 94°C for 20 s, annealing at 59°C for 20 s, and extension at 72°C for 20 s. The final extension was done at 72°C for 5 min. Amplification products were separated on non-denaturing 10% polyacrylamide gels and fragments were detected by ethidium bromide staining. Gels were documented using a UVI pro Platinum 1.1 System and UVI Band Map software (UVItec Ltd., Cambridge, UK).

### Statistical Analysis

Analysis of variance was carried out for each plant trait following Steel et al. (1997). Pair-wise comparison (LSD test at alpha 0.05) and correlation matrix were computed using XLSTAT 2010.

## Results

Complete breeding history (generations, breeding tasks, selection criteria, total number of crosses/plants/progenies/populations and number of selections at different stages) of the low phytate basmati rice mutants with improved germination and yield is presented in **Table 1**. Detailed results are as follows.

### Selection of Parents for Crossing Programme

**Figure 1:**
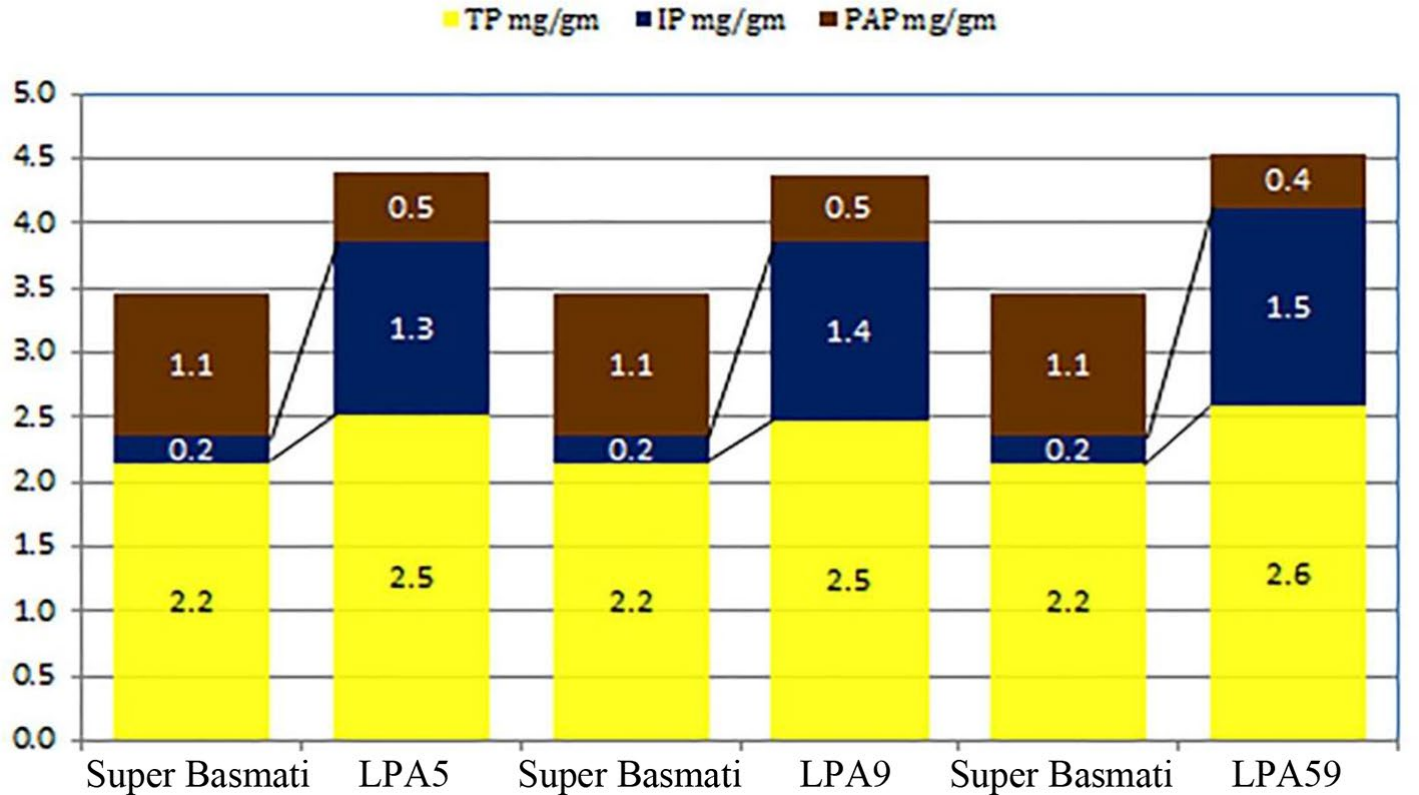
Quantitative analysis of total phosphorus (TP), phytic acid phosphorus (PAP), and inorganic phosphorus (IP) measured through HPLC analysis of low phytate mutants of parent cultivar Super basmati.

Low phytate mutants (Lpa5 and Lpa59) along with parent cultivar Super Basmati were selected for crossing program. These mutants showed 55% and 64% reduction in phytic acid respectively as compared to the parent cultivar Super Basmati. Quantitative analysis of total phosphorus (TP), phytic acid phosphorus (PAP) and inorganic phosphorus (IP) measured through HPLC analysis of these low phytate mutants along with parent cultivar Super Basmati is presented in **Figure 1**. Super Basmati showed 2.2 mg/g of TP, 0.2 mg/g IP and 1.1 mg/g PAP. Whereas PAP was reduced to 0.5 mg/g in Lpa5 and Lpa9 mutants as compared to 1.1 mg/g in the parent cultivar. Minimum PAP (0.4 mg/g) was observed in mutant Lpa59. However, these mutants and their progenies showed poor germination (about 15%) and yield in comparison with Super Basmati. Therefore, these low phytate mutants were selected and crossed with the parental variety to improve their yield and germination. Selection of parent cultivar (Super Basmati) was based on its prime position among the basmati cultivars of the country.

### Development and Screening of F_2:3_ and F_3:4_ Generation by the Colorimetric Assay Technique

During 2012–13, from 72 plants of the previous generation, F_2:3_ generations comprising 337 plants were developed and screened using the colorimetric assays. The assays showed these plants segregating for the low phytate trait. With respect to the low phytate trait the whole F_2:3_ populations were divided into three categories i.e., homozygous low phytate (226 plants), segregating low phytate (65) and non-low phytate (46).

**Figure 2:**
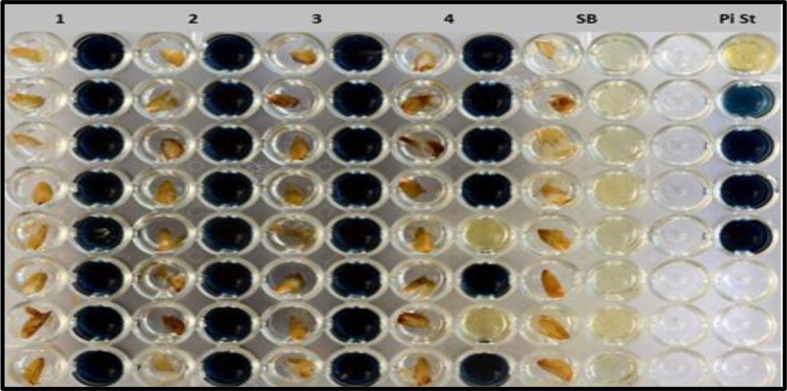
Colorimetric assay for the detection of low phytate trait including pure low phytate (1–3), segregating lines (4), Super Basmati (SB) and inorganic phosphorus standard (Pi St).

During 2013-14, in the F_3:4_ generation, two hundred and ninety one single plant progenies (226 homozygous and 65 heterozygous for low phytate trait) were harvested and screened by the colorimetric assay. Out of 291 lines, 86 were found to be homozygous for the low phytate trait, 71 were found to be segregating, and 134 were detected as non-low phytate or wild type. All three categories are shown in **Figure 2** and data are presented in **Table 2**. Wild type progenies were rejected and only homozygous and heterozygous progenies were selected for further evaluation of their germination and other desirable traits.

**Table 2:** Variation for low phytate trait and germination in F_2:3_ and F_3:4_ generations of crosses between low phytate mutants and wild type parent super basmati.

Sr. No.	Genotypes	No. of plants screened (F_2:3_)	No. of plants screened (F_3:4_)	Maximum Germination (%)
1	Homozygous	226	86	24
2	Heterozygous	65	71	70
3	Non Lpa lines	46	134	90
	Total	337	291	

### Germination Assessment of F_2:3_ Populations Under Field and Controlled Conditions

**Figure 3:**
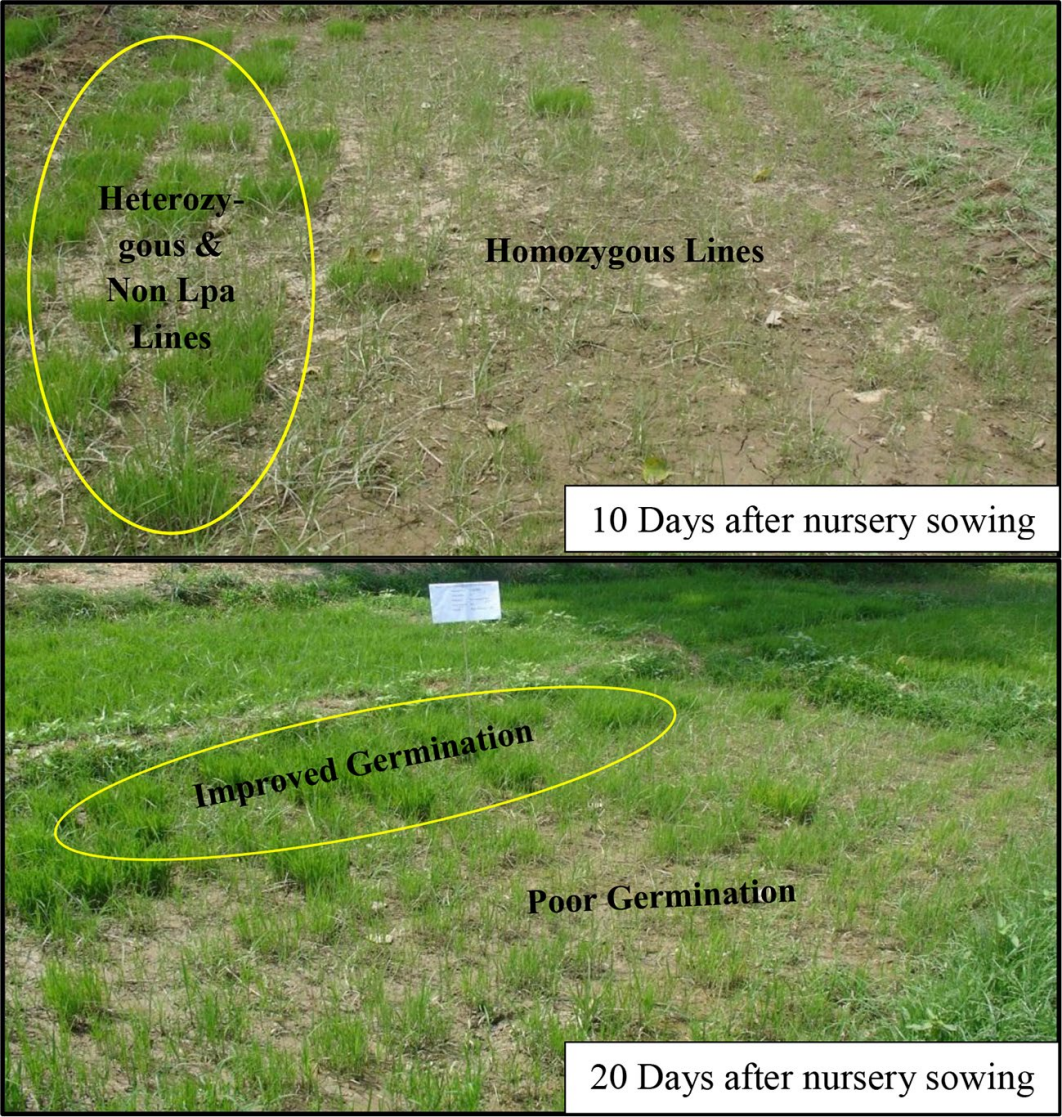
Germination tests of low phytate recombinants under field condition.

During 2012-13, field nurseries of F_2:3_ generations of low phytate lines were grown and observations were made on germination performance of the populations which comprised about 337 single plants. Results of the germination tests are presented in **Table 2** and the differences observed in germination are illustrated in **Figure 3**. The population showed clear segregation for germination behavior. Those plants which showed better germination were tested for the low phytate trait from the remaining seeds in the laboratory and it was observed that most of the recombinants with better germination are heterozygous for low phytate trait. However, three plants (Plant No. 5, 6, 30) showed relatively better germination (about 10%) and seedling vigor than parental low phytate lines and these plants were also found to be homozygous for Lpa trait. The germination tests were performed in the field nursery where conditions are generally non-uniform. Therefore, during 2013–14, further germination tests, with some additional selected lines, were performed under controlled conditions. Eight single plant lines, given in **Supplementary Table S1**, were tested for germination potential under controlled conditions. Among the tested lines, Lpa5, Lpa6, Lpa7, and Lpa30 showed relatively better germination over rest of the lines. However, all these lines exhibited poor germination compared to the parental cultivar Super Basmati.

### Germination and Grain Quality Parameters Assessment of Selected Low Phytate Recombinants (F_3:4_ Generation)

During the year 2014-15, selections for relatively high yielding low phytate lines were continued and 38 lines were selected on the basis of better plant type, grain quality and presence of the low phytate trait. The results of the colorimetric assays, grain quality parameters, cooking characteristics, and germination tests under controlled conditions are presented in **Supplementary Table S2**. The germination percentage of these 38 lines ranged from 4% to 42% which suggested the presence of some other minor genes which segregate along with the low phytate trait. Maximum germination was shown by progeny number Lpa101-4. All recombinants were found homozygous for Lpa trait. Grain quality and cooking characteristics of these selected recombinants were also comparable with the parent cultivar. Thus, the results indicated that breeding for low phytate trait through induced mutagenesis and cross breeding does not compromise the superior grain quality, cooking characteristics and aroma which is specialty of basmati rice.

### Raising and Screening of BC_1_F_2:3_ Generation

During 2013–14, as a second strategy to improve germination and yield, back cross generations (BC_1_F_1:2_) were developed by crossing parent variety Super Basmati with the F_1_ generation of low phytate basmati rice mutants. BC_1_F_1:2_ generations were sown in the field to develop BC_1_F_2:3_ seed. All BC_1_F_2:3_ populations were raised in the field during kharif 2013-14. Keeping in view desirable plant attributes, 9, 38, 11, and 53 plants were selected from four populations Lpa5 × Super Basmati × Super Basmati; Super Basmati × Lpa5 × Super Basmati; Lpa59 × Super Basmati × Super Basmati; and Super Basmati × Lpa59 × Super Basmati, respectively.

### Performance of the 1^st^ BC_1_F_2:3_ Populations (Lpa5 × Super Basmati × Super Basmati)

This cross involved nine plants. The results of this cross are presented in **Supplementary Table S3**. Colorimetric assays identified all these lines as heterozygous. The plant height ranged from 129 cm to 146 cm, productive tillers per plant ranged from 14 to 18, panicle length between 29 cm and 31.5 cm, primary branches per panicle 9 to 11, fertility percentage 72% to 96.35% and yield per plant ranged between 21.6 g to 29.6 g. As compared to control all lines were proved to be high yielder.

### Performance of the 2^nd^ BC_1_F_2:3_ Population (Super Basmati × Lpa5 × Super Basmati)

This cross included 38 plants. The results of this cross are presented in **Supplementary Table S4**. Colorimetric assays revealed one plant as homozygous (Lpa141), nine plants heterozygous, and 27 plants as wild type/non-low phytate. Data of one plant is missing but it was planted in the field. The plant height ranged from 118 cm to 140 cm, productive tillers per plant 9 to 17, panicle length 19 cm to 30.5 cm, primary branches per panicle 9 to 11, fertility percentage 89% to 96.55%, and yield per plant 11.6 g to 23.8 g. Three plants (Lpa138, Lpa165, and Lpa166) were found to be higher yielding than control Super Basmati.

### Performance of the 3^rd^ BC_1_F_2:3_ Populations (Lpa59 × Super Basmati × Super Basmati)

This cross involved 11 plants. The results of this cross are presented in **Supplementary Table S5**. Based on colorimetric assays, all the plants were found to be heterozygous for the low phytate trait. Data on yield and related components showed that plant height ranged between 120 cm to 134 cm, productive tillers per plant 11 to 21, panicle length 25 cm to 31 cm, and primary branches per panicle 9 to 12, and fertility percentage 87.9 to 95.39. Paddy yield per plant ranged between 16 g to 31.2 g. Five plants (Lpa70, Lpa71, Lpa72, Lpa73, and Lpa79) were found to be high yielding than control Super Basmati.

### Performance of the 4^th^ BC_1_F_2:3_ Populations (Super Basmati × Lpa59 × Super Basmati)

This population involved 53 plants. The results of this cross are presented in **Supplementary Table S6**. Colorimetric analysis revealed that 23 plants were heterozygous. Twenty nine plants were non low phytate wild type. Only one plant (Lpa205) was homozygous for the low phytate trait. Data on yield and yield components revealed that plant height ranged between 113 cm to 142 cm, primary tillers per plant 8 to 22, panicle length 27 cm to 38 cm, primary branches per panicle 8 to 16, fertility percentage 83.23% to 95.8%, and yield per plant 8.4 g to 31 g. Thirteen progenies (Lpa178, Lpa180, Lpa186, Lpa188, Lpa190, Lpa195, Lpa198, Lpa199, Lpa202, Lpa204, Lpa208, Lpa212, and Lpa214) were proved to be higher yielding than parental cultivar.

### Performance of the BC_1_F_3:4_ Progenies

From 111 single plant progenies of four BC_1_F_2:3_ populations, about 109 plants were selected and raised in the field (**Supplementary Table S7**). At maturity, selections within the segregating progenies of back cross populations were continued based on desirable plant attributes and homozygosity for the low phytate trait. Colorimetric assay of 109 single plant progenies was performed. Forty nine lines were found heterozygous for the low phytate trait, two homozygous and fifty eight were non Lpa. Lpa141-4 and Lpa205-4 progenies were found to be homozygous for low phytate trait within 2^nd^BC_1_F_3:4_ and 4^th^ BC_1_F_3:4_ populations, respectively.

### Field Performance of Selected Low Phytate Recombinants

**Figure 4:**
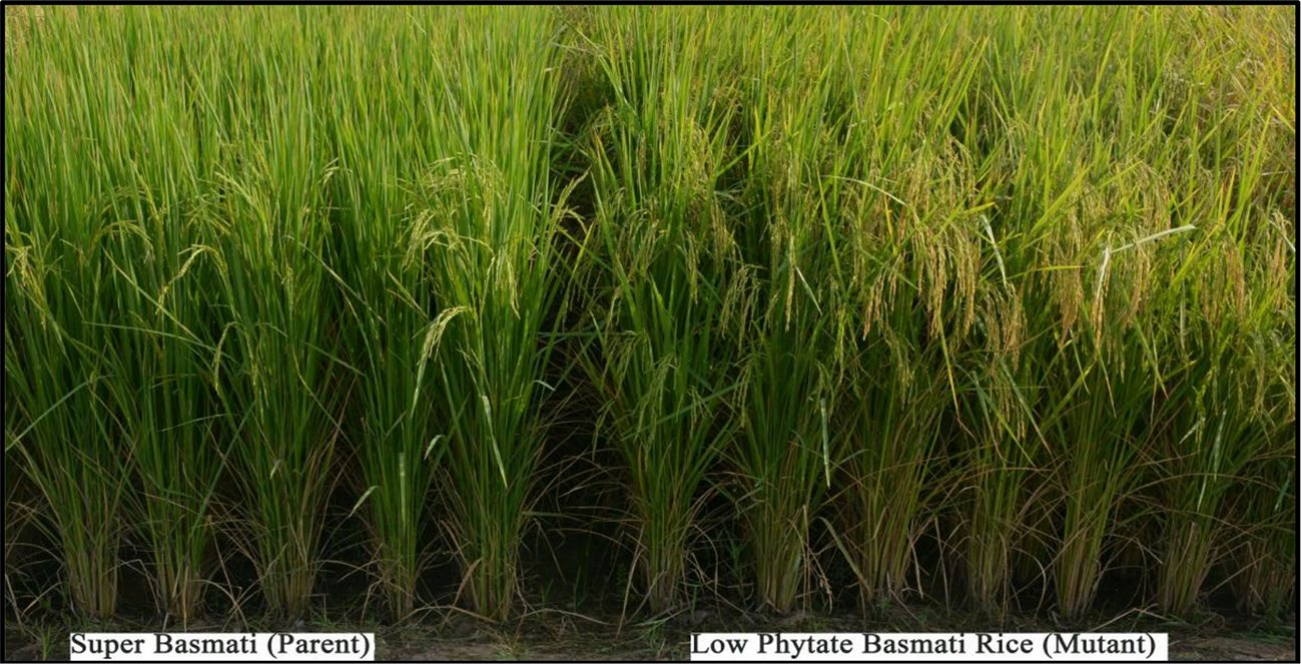
Low phytate mutant with improved germination and yield over parent variety (Super Basmati).

Based on desirable agronomic traits, better germination, yield, and homozygosity for the low phytate trait, 42 lines (38 from F_3:4,_ 02 BC_1_F_2:3,_ and 02 BC_1_F_3:4_) were selected for further evaluation in a field trial. During 2015-16, a preliminary yield trial of these homozygous low phytate selected lines, in an RCBD design was conducted. Analysis of variance showed highly significant differences among the progenies for plant height, panicle length, productive tillers per plant and yield per plant (gm) and data are presented in **Table 3**. In order to categorize the progenies, pair wise comparisons (LSD test) were performed and data are presented in **Table 4**. Pair wise comparison categorized all the 42 progenies into 13 groups with respect to plant height, 7 groups with respect to panicle length, 6 groups with respect to productive tillers per plant and 9 groups with respect to paddy yield per plant (gm). Seven lines, Lpa12-3 (18%), Lpa111-1 (17%), Lpa141 (15%), Lpa56-3 (13%), Lpa53-4 (12%), Lpa99-2 (12%), and Lpa205-4 (4%) produced better yield over the control cultivar Super Basmati. Values in bracket represent the level of yield improvement compared with the parental line. Trait association analysis for yield improvement was also performed for further selection of high yielding plants among the progenies. Analysis exhibited that paddy yield was significantly and positively correlated with primary branches per plant, panicle length, and productive tillers per plant (**Table 5**). Therefore, further selection based on these traits would be helpful to improve the paddy yield. A field view of the low phytate mutant having improved germination and yield over the parent variety (Super Basmati) is presented in Figure 4.

**Table 3:** Analysis of variance of yield and some associated traits in preliminary yield trial of selected low phytate lines under Faisalabad conditions.

SOV	df	Mean Squares					
		PBRP	F %age	PH (cm)	PL (cm)	PT/P	Y/P (gm)
R	2	1.68	1.15	3.26	0.38	5.24	55.03
T	42	1.67	19.75	46.05**	3.47**	19.62**	71.69**
Error	84	1.28	14.15	6.54	1.42	6.02	18.97

PBRP, Primary branches per plant; F, Fertility percentage; PH, Plant height; PL, Panical Length; PT/P, Productive tillers per plant; Y/P, Yield per plant.

**Significant at 0.01 probability level.

**Table 4:** Pairwise comparison of yield and associated traits in 42 advance low phytate lines.

Sr. No	Progeny No.	PBRP	F%	PH (cm)	PL (cm)	PTL	Y/P (gm)	% change (over check)
1	Lpa6-3	9.3^ab^	90.0^abc^	125.3^bcdefgh^	28.7^cdef^	19.6^ab^	12.8^bcdefghi^	-21
2	Lpa7-3	9.6^ab^	91.0^abc^	124.3^cdefghij^	28.3^defg^	17.0^bcde^	16.1^bcdefgh^	-1
3	Lpa10-2	9.0^ab^	88.3^abc^	125.6^bcdefgh^	28.3^defg^	16.6^bcdef^	9.5^efghi^	-42
4	Lpa12-3	9.3^ab^	89.7^abc^	132.6^a^	28.0^efg^	22.3^a^	19.2^a^	18
5	Lpa12-4	9.3^ab^	91.3^abc^	127.0^bcdef^	28.6^cdef^	16.3^bcdef^	12.7^bcdefghi^	-22
6	Lpa53-1	9.0^ab^	86.6^abc^	127.6^abcde^	30.3^abcde^	18.6^abcd^	14.5^bcdefghi^	-11
7	Lpa53-4	9.3^ab^	84.7^c^	127.6^abcde^	30.0^abcdef^	16.6^bcdef^	13.0^bcdefghi^	-20
8	Lpa55-1	9.3^ab^	85.7^bc^	124.6^bcdefghi^	30.0^abcdef^	18.6^abcd^	18.3^bcdef^	12
9	Lpa55-2	6.3^c^	92.0^abc^	125.3^bcdefgh^	29.6^abcdef^	15.3^bcdef^	9.9^cdefghi^	-39
10	Lpa55-4	8.7^abc^	93.0^ab^	125.0^bcdefghi^	29.3^abcdef^	19.6^ab^	15.3^bcdefgh^	-6
11	Lpa56-1	9.3^ab^	87.3^abc^	130.0^ab^	31.0^abc^	16.0^bcdef^	15.1^bcdefgh^	-7
12	Lpa56-3	11.0^a^	92.0^abc^	129.33^abc^	31.6^a^	18.6^abcd^	18.4^bcdef^	13
13	Lpa56-4	9.0^ab^	92.7^abc^	127.6^abcde^	30.0^abcdef^	13.6^def^	9.5^efghi^	-42
14	Lpa63-3	9.0^ab^	88.0^abc^	126.3^bcdef^	30.3^abcde^	17.0^bcde^	13.0^bcdefghi^	-20
15	Lpa66-3	8.3^bc^	94.0^a^	127.7^abcde^	29.0^bcdef^	14.3^cdef^	9.3^fghi^	-43
16	Lpa99-1	8.3^bc^	90.7^abc^	128.0^abcd^	28.3^defg^	14.3^cdef^	8.07^ghi^	-50
17	Lpa99-2	9.3^ab^	92.0^abc^	125.3^bcdefgh^	28.6^cdef^	17.0^bcde^	18.3^bcdef^	12
18	Lpa101-1	9.3^ab^	92.3^abc^	125.3^bcdefgh^	29.6^abcdef^	13.6^def^	8.3^ghi^	-49
19	Lpa101-3	9.6^ab^	94.0^a^	123.6^defghijk^	29.6^abcdef^	12.6^ef^	8.3^ghi^	-49
20	Lpa101-4	9.3^ab^	90.7^abc^	121.6^fghijkl^	30.6^abcd^	11.6^f^	7.6^hi^	-53
21	Lpa111-1	10.3^ab^	92.3^abc^	127.6^abcde^	31.3^ab^	17.3^abcde^	19.07^bcd^	17
22	Lpa122-1	10.0^ab^	89.0^abc^	119.6^ijkl^	29.6^abcdef^	22.3^a^	11.5^cdefghi^	-29
23	Lpa122-4	10.3^ab^	90.7^abc^	121.6^fghijkl^	30.3^abcde^	19.0^abc^	9.7^efghi^	-40
24	Lpa123-2	9.3^ab^	88.3^abc^	125.0^bcdefghi^	29.0^bcdef^	13.6^def^	7.9^ghi^	-52
25	Lpa123-3	9.0^ab^	94.3^a^	128.0^abcd^	27.6^fg^	12.6^ef^	7.5^hi^	-54
26	Lpa124-1	9.0^ab^	85.0^bc^	127.6^abcde^	28.6^cdef^	13.6^def^	11.3^cdefghi^	-31
27	Lpa138-1	10.0^ab^	88.6^abc^	119.0^jkl^	29.6^abcdef^	16.6^bcdef^	8.0^ghi^	-51
28	Lpa138-2	9.6^ab^	88.6^abc^	126.0^bcdefg^	29.6^abcdef^	14.0^cdef^	5.5^i^	-66
29	Lpa138-3	9.6^ab^	87.0^abc^	110.3^m^	26.0^g^	12.6^ef^	8.3^ghi^	-49
30	Lpa144-4	9.3^ab^	91.0^abc^	127.0^bcdef^	29.3^abcdef^	16.6^bcdef^	10.4^cdefghi^	-36
31	Lpa154-2	10.0^ab^	89.6^abc^	122.6^defghijkl^	29.3^abcdef^	13.6^def^	7.0^hi^	-57
32	Lpa166-1	10.0^ab^	89.0^abc^	128.0^abcd^	29.3^abcdef^	15.0^bcdef^	14.0^bcdefghi^	-14
33	Lpa166-3	8.6^abc^	84.6^c^	125.7^bcdefgh^	28.6^cdef^	14.3^cdef^	10.3^cdefghi^	-37
34	Lpa169-4	9.0^ab^	86.3^abc^	124.0^cdefghij^	31.0^abc^	16.3^bcdef^	8.7^ghi^	-47
35	Lpa174-2	10.3^ab^	85.3^bc^	120.3^hijkl^	30.6^abcd^	15.6^bcdef^	13.3^bcdefghi^	-18
36	Lpa174-4	9.0^ab^	89.0^abc^	120.7^ghijkl^	29.6^abcdef^	13.0^ef^	8.0^ghi^	-51
37	Lpa200-1	9.3^ab^	91.0^abc^	122.0^fghijkl^	28.6^cdef^	15.3^bcdef^	8.6^ghi^	-47
38	Lpa200-2	10.3^ab^	89.3^abc^	124.3^cdefghij^	28.6^cdef^	15.0^bcdef^	9.7^defghi^	-40
39	Lpa141	10.0^ab^	89.3^abc^	122.3^efghijkl^	30.0^abcdef^	16.6^bcdef^	18.8^bcde^	15
40	Lpa205	10.3^ab^	88.0^abc^	123.6^defghijk^	30.6^abcd^	14.0^def^	11.9^cdefghi^	-27
41	Lpa141-4	10.0^ab^	91.3^abc^	118.33^kl^	29.3^abcdef^	14.3^cdef^	14.7^bcdefghi^	-10
42	Lpa205-4	9.6^ab^	88.6^abc^	122.3^efghijkl^	29.6^abcdef^	12.6^ef^	17.0^bcdefg^	4
Super basmati (check)		9.3^ab^	89.7^abc^	118.0^l^	28.3^defg^	12.6^ef^	16.3^bcdefghi^	-

Means followed by the same letter in the same column are not significantly different (LSD test, α=5%).

**Table 5:** Correlation Matrix of some yield associated traits in 42 low phytate lines of basmati rice.

Correlation	PBPP	F%	PH (cm)	PL (cm)	PT/P
F%	0.0062				
*Prob*	*0.9441*				
PH (cm)	-0.1473	0.1105			
*Prob*	*0.0958*	*0.2125*			
PL (cm)	0.1914	0.0737	0.228**		
*Prob*	*0.0298*	*0.4065*	*0.0093*		
PT/P	0.1221	0.02	0.1899*	0.1557	
*Prob*	*0.1682*	*0.8223*	*0.0311*	*0.0781*	
Y/P(gm)	0.2765**	0.0287	0.2081	0.3382**	0.4294**
*Prob*	*0.0015*	*0.7469*	*0.0179*	*0.0001*	*0.0001*

*,**Correlation significant at 0.05 and 0.01 probability levels, respectively.

### Identification and Characterization of Lpa Mutations Using Molecular Markers

PCR conditions for the use of molecular markers i.e., SSR, Indel and CAPS were optimized. The Lpa mutants along with parent Super Basmati were characterized using SSR, CAPS, and InDel markers. Representative gels for SSR markers are presented as **Supplementary Figure S1**. Little polymorphism was detected among tested Lpa mutants and parental genotype for most of the screened primers. Marker RM-55 detected polymorphism among Lpa mutants and parental genotype (**Supplementary Figure S1**). The parental genotype, Super Basmati (WT), and Lpa mutants were characterized using recently reported functional molecular markers for five Lpa mutant alleles of three different genes.

ZB-Lpa resulted from allelic mutation of a sulfate transporter (OsST) gene; the mutation is a 6 bp deletion. Because the Z9B-Lpa allele is 6 bp shorter than the wild type (98 bp), an insertion–deletion marker was developed by using primer pair P5F/R; the amplicons of Z9B-Lpa (92 bp) could be easily differentiated from those of WT and Lpa using polyacrylamide gel electrophoresis. For our tested samples homozygous WT allele of 98 bp was detected in all parent and mutant samples (**Supplementary Figure S2**) indicating absence of Z9B-Lpa allele in tested mutants.

The base pair change (C/G to T/A) in XS-Lpa abolished the restriction site of *Hae*III in the OsMRP5 gene, which enabled the development of a CAPS marker for distinguishing the XS-Lpa allele from the WT allele. By using specific primer pair, a fragment of 1,166 bp was amplified for both the WT and XS-Lpa allele (**Supplementary Figure S3**). However, the WT amplicons could be digested with *Hae*III into two fragments (721 and 445 bp), while those of XS-Lpa could not, and thus plants of different genotypes, viz. homozygous WT, homozygous XS-Lpa, and heterozygous, could be easily distinguished through length polymorphism analysis of digested amplicons. In the present study, after digestion with *Hae*III, two fragments i.e., 721 and 445 bp showing a homozygous WT allele were detected in parent genotype (WT) and in tested mutants (**Supplementary Figure S3**). Results thus indicated the absence of XS-Lpa mutation in the OsMRP5 gene in tested mutants.

## Discussion

Genetic biofortification or breeding of crops for increased level of nutrients and vitamins or decreased levels of “anti-nutrients” such as phytic acid signifies an approach to combat mineral deficiency. Bio-fortification of crops is recognized as a cost effective and environment friendly solution to combat the problem. The world is now focusing on bio-fortification interventions, such as by breeding low phytate crops, to fulfill nutritional requirements without bearing additional costs. Development of Golden rice to combat vitamin-A deficiency and the development of low phytate maize, rice, soybean, wheat, and barley by USDA are important examples to address the mineral deficiencies by bio-fortification intervention (Raboy, 2002; Dorsch et al., 2003; Raboy, 2007). The first low phytate genotypes were isolated in maize using colorimetric assays of individual seeds sampled from a chemically-mutagenized population with a direct test for reduced phytic acid (Raboy et al., 2000). Studies of these first mutants revealed that seed total P was not greatly changed; phytic acid phosphate reductions were matched by increases in inorganic phosphate. Testing for inorganic P as compared with phytic acid is technically simple, fast and inexpensive. Also, the relative increase in inorganic P in low phytate genotypes is many-fold as compared to wild type. Therefore, high inorganic P phenotype of low phytate genotypes provides for a high-throughput screen for low phytate types. Using this high-throughput test, additional low phytate genotypes have been isolated in maize and in a number of additional species including barley, rice, wheat, soybean, common bean, and field pea (Cichy and Raboy, 2009; Warkentin et al., 2012). These studies have shown that when foods are prepared with low phytate types as compared with normal phytate types, mineral availability (iron, zinc and calcium) increases from 30% to 50%. Lonnerdal et al. (2011) while studying the nutritional aspects of the “rat pup” model found that zinc absorption from low phytate rice was increased 70% in contrast with wild-type. In the present study, we screened a mutagenized M_2_ generation of cultivar “Super basmati” and found some low phytate mutants in basmati rice. These mutants had issues of low germination and yield. Therefore, the basic objective of the current project was to improve the germination and yield of these low phytate mutants by hybridization and backcross breeding. Hybridization and backcross breeding are important tools in the hands of breeders to get the desirable recombinants of the targeted trait. Both approaches are used to transfer the gene (s) of interest. The objective of the present study was to transfer the low phytate trait into the adapted high yielding variety Super basmati. For this purpose, selected low phytate mutants were crossed with cultivar Super Basmati to select for low phytate recombinants. Different segregating generations F_2:3_ and F_3:4_ were developed to select the plants with desirable traits. These plants were further screened by the colorimetric assays to detect the low phytate mutation. Since recombination is a random event, therefore during the present study two approaches (screening of F_2:3_ & F_3:4_ and BC_1_F_2:3_ & BC_1_F_3:4_) were opted to address the objective. Since, rice is self-pollinated crop (Matsui and Kagata, 2003), therefore, clear segregation patterns, for the traits of interest, were observed within filial generations. Back crossing with the adapted high yielding variety resulted in the improvement of yield along with desired traits under consideration. The philosophy behind this back crossing was to recover such recombinants which have comparable yield and germination as the control Super Basmati along with the low phytate trait. Since low phytate trait is a recessive mutation (Larson et al., 2000; Raboy et al., 2000; Wilcox et al., 2000), maximum segregating material was developed through hybridization and backcross breeding to maximize the chances of desirable recombinants. Out of four BC_1_F_2:3_ populations, 111 plants, with desirable plant attributes, were selected. Colorimetric assays revealed segregation for the low phytate trait. Out of 111 plants, only two plants Lpa141 and Lpa205 were found to be homozygous for the low phytate trait. The low frequency of pure low phytate lines in BC_1_F_2:3_ populations might be due to the recessive behavior of the low phytate trait or more likely that there are a number of genes affecting this trait. Alternatively, there may be segregation distortion against the homozygote. Therefore, further selection of the plants within the segregating low phytate BC_1_F_2:3_ lines might be useful to recover further desired recombinants.

Most primary Lpa mutants often feature inferior grain yield, reduced seed viability or field emergence compared to their respective wild-type parents and thus further improvement is needed before new Lpa crops can be put into practical use (Raboy, 2009). Zhao et al. (2008a) observed significantly low grain yield (12.5%–25.6%) in four to five low phytate mutants of rice, and it was mainly due to a reduction in grain weight, varying from 5.4% to 10.7%. They also observed significantly lower vigour index in all mutant lines as compared to their parents, with reduction of 7.8%–26.3%. However, it has been reported that grain yield and field emergence can be improved through cross and selection breeding in rice (Zhou et al., 2019). Cross breeding and marker assisted selection were also used in soybean to improve germination and yield of low phytate mutants (Spear and Fehr, 2007) and indeed two barley Lpa cultivars, Clearwater, and CDC Lophy-1, have already been released for commercial production (Bregitzer et al., 2008; Rossnagel et al., 2008). The identification of the Lpa gene and development of allele-specific markers are of importance not only for breeding Lpa varieties, but also for advancing genetics and genomics of phytic acid biosynthesis in rice and other plant species. Some genes have been identified to be potentially involved in phytic acid biosynthesis in rice (Josefsen et al., 2007; Suzuki et al., 2007), but it is not yet known whether mutation of these genes could result in reduction of phytic acid in rice seeds like the MIPS1 gene (Kuwano et al., 2006; Kuwano et al., 2009). For this reason, efforts were made to characterize our Lpa mutant lines through DNA based markers. To check the genetic variability among parent genotype and mutants lines SSR markers were applied and some of them produced polymorphic profiles. However, these previously reported markers were not breeder friendly having several limitations. Since Lpa is a seed trait, closely linked or functional molecular markers are extremely useful for breeding new Lpa crop varieties. Previous studies have identified several causative Lpa mutations, e.g., two allelic mutations (XQZ-Lpa and KBNT-Lpa) of LOC_Os02g57400 (Kim et al., 2008; Zhao et al., 2008b), one mutation (XS-Lpa) of the myo-inositol kinase (OsMIK) gene (Kim et al., 2008), and two allelic mutations (Z9B-Lpa and MH-Lpa) of OsMRP5 (Xu et al., 2009). To enable marker assisted breeders to efficiently utilize the Lpa mutations in rice breeding programs, highly reproducible and breeder-friendly functional molecular markers are required. Very recently, such highly reproducible and breeder-friendly functional molecular markers were developed for five Lpa mutant alleles of three different genes and their utility was assessed for marker-assisted selection of Lpa plants (Tan et al., 2013). We were able to establish and then assess the usability of these recently reported molecular markers for marker-assisted selection of the Lpa trait in rice. In the present study, markers for ZB-Lpa allelic mutation (6 bp deletion) of a sulfate transporter (OsST) gene and a single base pair change (C/G to T/A) in XS-Lpa that abolished the restriction site of *Hae*III in the OsMRP5 gene were successfully applied to rice mutants. For both genes, WT alleles were detected in parent and mutants. The functional molecular markers were highly reproducible and breeder-friendly for the Lpa trait. Therefore, after screening out these mutant alleles, there is the possibility of other mutations or novel alleles in the tested rice mutants that needs to be identified and then fine mapped for subsequent utilization.

## Conclusion

In conclusion, through induced mutagenesis, Lpa mutants using cultivar Super Basmati were developed, verified through calorimetric assays followed by HPLC analysis, which indicated that mutants have 54%–63% reduction in phytic acid. To our knowledge this is the first report of LPA rice mutants in a basmati background (aromatic). Moreover, through hybridization and back cross breeding, recombinants having better germination and yield, over the parental low phytate mutants were also developed. For molecular characterization of Lpa mutants, different marker systems (SSR, Lpa1-CAPS and Lpa1-InDel) were applied including recently reported functional molecular markers for five Lpa mutant alleles of three different genes. Wild Type alleles were detected for reported Lpa mutant alleles in parent and mutants. Therefore, there is strong possibility of new mutations or novel alleles in our rice mutants that needs to be identified and then fine mapped for subsequent utilization.

## Data Availability Statement

All datasets generated for this study are included in the article/**Supplementary Material**.

## Author Contributions

Z-u-Q was involved in giving basic idea and planning of the study. AH conducted lab experiment relevant to marker assisted selection. Z-u-Q and MAs executed field experiments. MAk and MR helped in performing different assays involved in the study and execution of field trial. Z-u-Q, AH, and MR also contributed in data analysis, manuscript writing and finalization of draft. MR critically analyzed and corrected the manuscript.

## Conflict of Interest

The authors declare that the research was conducted in the absence of any commercial or financial relationships that could be construed as a potential conflict of interest.
