# Supplementary material for: Development and Molecular Characterization of Low Phytate Basmati Rice Through Induced Mutagenesis, Hybridization, Backcross, and Marker Assisted Breeding
Source: Front Plant Sci. 2019 Nov 26;10:1525. doi: 10.3389/fpls.2019.01525 (PMC6901921; doi:10.3389/fpls.2019.01525)
Supplement: Supplementary file 1 [file DataSheet_1.docx]

Table S1: Germination of recombinant Lpa lines selected in the F_2:3_ generation of recombinant progenies during 2012-13

| **Sr. No** | **Progeny No** | **Total seeds** | **Seeds germinated** | **Germination %age** |
| --- | --- | --- | --- | --- |
| 1 | Lpa5 | 50 | 19 | 38 |
| 2 | Lpa6 | 50 | 19 | 38 |
| 3 | Lpa7 | 50 | 14 | 28 |
| 4 | Lpa30 | 50 | 23 | 46 |
| 5 | Lpa58 | 50 | 3 | 6 |
| 6 | Lpa73 | 50 | 4 | 8 |
| 7 | Lpa114 | 50 | 9 | 18 |
| 8 | Lpa132 | 50 | 1 | 2 |
| Super Basmati (Control) | | 50 | 32 | 64 |

^Lpa: Low phytic acid^

Table S2: Germination and grain quality assessment of recombinant Lpa lines selected in the F_3:4_ generation of recombinant progenies under controlled conditions during 2014-15

| **Sr. No.** | **Progeny** | **G %** | **Lpa test** | **PDL (mm)** | **PDW (mm)** | **PDL/PDW** | **GL**  **(mm)** | **GW**  **(mm)** | **GL/GW** | **CGL**  **(mm)** | **ER** | **HRR**  **(%)** | **B (%)** | **Aroma** |
| --- | --- | --- | --- | --- | --- | --- | --- | --- | --- | --- | --- | --- | --- | --- |
| 1 | Lpa6-3 | 22 | Homo | 10.56 | 2 | 5.28 | 7.42 | 1.50 | 4.95 | 12.9 | 1.74 | 56 | 4 | + |
| 2 | Lpa7-3 | 28 | Homo | 10.72 | 2 | 5.36 | 7.47 | 1.50 | 4.98 | 13.5 | 1.81 | 53 | 6 | + |
| 3 | Lpa10-2 | 4 | Homo | 10.74 | 2 | 5.37 | 7.47 | 1.50 | 4.98 | 13.5 | 1.81 | 53 | 6 | + |
| 4 | Lpa12-3 | 22 | Homo | 10.60 | 2 | 5.30 | 7.39 | 1.50 | 4.93 | 13.5 | 1.83 | 56 | 4 | + |
| 5 | Lpa12-4 | 40 | Homo | 10.68 | 2 | 5.34 | 7.39 | 1.50 | 4.93 | 13.5 | 1.83 | 57 | 3 | + |
| 6 | Lpa53-1 | 26 | Homo | 10.80 | 2 | 5.40 | 7.46 | 1.50 | 4.97 | 13.5 | 1.81 | 57 | 4 | + |
| 7 | Lpa53-4 | 18 | Homo | 10.80 | 2 | 5.40 | 7.46 | 1.50 | 4.97 | 13.5 | 1.81 | 56 | 5 | + |
| 8 | Lpa55-1 | 38 | Homo | 11.00 | 2 | 5.50 | 7.47 | 1.50 | 4.98 | 13.5 | 1.81 | 58 | 3 | + |
| 9 | Lpa55-2 | 18 | Homo | 10.90 | 2 | 5.46 | 7.47 | 1.50 | 4.98 | 13.5 | 1.81 | 59 | 3 | + |
| 10 | Lpa55-4 | 12 | Homo | 10.96 | 2 | 5.48 | 7.47 | 1.50 | 4.98 | 13.5 | 1.81 | 59 | 3 | + |
| 11 | Lpa56-1 | 24 | Homo | 11.00 | 2 | 5.50 | 7.47 | 1.50 | 4.98 | 13.5 | 1.81 | 58 | 3 | + |
| 12 | Lpa56-3 | 32 | Homo | 11.00 | 2 | 5.50 | 7.47 | 1.50 | 4.98 | 13.5 | 1.81 | 52 | 7 | + |
| 13 | Lpa56-4 | 40 | Homo | 10.92 | 2 | 5.46 | 7.47 | 1.50 | 4.98 | 13.5 | 1.81 | 51 | 7 | + |
| 14 | Lpa63-3 | 38 | Homo | 10.96 | 2 | 5.48 | 7.47 | 1.50 | 4.98 | 13.5 | 1.81 | 52 | 6 | + |
| 15 | Lpa66-3 | 22 | Homo | 10.96 | 2 | 5.48 | 7.47 | 1.50 | 4.98 | 13.5 | 1.81 | 53 | 6 | + |
| 16 | Lpa99-1 | 22 | Homo | 10.96 | 2 | 5.48 | 7.47 | 1.50 | 4.98 | 13.5 | 1.81 | 57 | 3 | + |
| 17 | Lpa99-2 | 18 | Homo | 10.92 | 2 | 5.46 | 7.47 | 1.50 | 4.98 | 13.5 | 1.81 | 53 | 5 | + |
| 18 | Lpa101-1 | 26 | Homo | 10.40 | 2 | 5.21 | 7.39 | 1.50 | 4.93 | 12.7 | 1.72 | 55 | 5 | + |
| 19 | Lpa101-3 | 30 | Homo | 10.40 | 2 | 5.21 | 7.39 | 1.50 | 4.93 | 12.7 | 1.72 | 53 | 6 | + |
| 20 | Lpa101-4 | 42 | Homo | 10.40 | 2 | 5.22 | 7.39 | 1.50 | 4.93 | 12.9 | 1.75 | 56 | 3 | + |
| 21 | Lpa111-1 | 24 | Homo | 10.30 | 2 | 5.16 | 7.34 | 1.50 | 4.89 | 12.5 | 1.70 | 57 | 3 | + |
| 22 | Lpa122-1 | 14 | Homo | 10.40 | 2 | 5.22 | 7.36 | 1.50 | 4.91 | 13.5 | 1.83 | 56 | 4 | + |
| 23 | Lpa122-4 | 18 | Homo | 10.40 | 2 | 5.22 | 7.36 | 1.50 | 4.91 | 13.5 | 1.83 | 56 | 3 | + |
| 24 | Lpa123-2 | 28 | Homo | 10.30 | 2 | 5.17 | 7.36 | 1.50 | 4.91 | 12.5 | 1.70 | 57 | 3 | + |
| 25 | Lpa123-3 | 34 | Homo | 10.20 | 2 | 5.02 | 7.26 | 1.50 | 4.84 | 12.4 | 1.71 | 54 | 6 | + |
| 26 | Lpa124-1 | 14 | Homo | 10.90 | 2 | 5.43 | 7.47 | 1.50 | 4.98 | 13.5 | 1.81 | 54 | 6 | + |
| 27 | Lpa138-1 | 28 | Homo | 10.30 | 2 | 5.14 | 7.26 | 1.50 | 4.84 | 12.8 | 1.76 | 53 | 6 | + |
| 28 | Lpa138-2 | 14 | Homo | 10.40 | 2 | 5.19 | 7.34 | 1.50 | 4.89 | 12.9 | 1.76 | 53 | 7 | + |
| 29 | Lpa138-3 | 6 | Homo | 10.20 | 2 | 5.12 | 7.26 | 1.50 | 4.84 | 12.7 | 1.75 | 53 | 7 | + |
| 30 | Lpa144-4 | 14 | Homo | 10.70 | 2 | 5.36 | 7.38 | 1.50 | 4.92 | 12.3 | 1.67 | 58 | 3 | + |
| 31 | Lpa154-2 | 22 | Homo | 10.40 | 2 | 5.22 | 7.26 | 1.50 | 4.84 | 12.6 | 1.74 | 55 | 5 | + |
| 32 | Lpa166-1 | 38 | Homo | 11.10 | 2 | 5.54 | 7.47 | 1.50 | 4.98 | 13.7 | 1.83 | 54 | 5 | + |
| 33 | Lpa166-3 | 10 | Homo | 11.00 | 2 | 5.52 | 7.47 | 1.50 | 4.98 | 13.6 | 1.82 | 55 | 5 | + |
| 34 | Lpa169-4 | 20 | Homo | 10.60 | 2 | 5.29 | 7.39 | 1.50 | 4.93 | 12.9 | 1.75 | 54 | 5 | + |
| 35 | Lpa174-2 | 30 | Homo | 10.70 | 2 | 5.34 | 7.39 | 1.50 | 4.93 | 12.9 | 1.75 | 57 | 3 | + |
| 36 | Lpa174-4 | 32 | Homo | 10.80 | 2 | 5.40 | 7.39 | 1.50 | 4.93 | 12.9 | 1.75 | 58 | 3 | + |
| 37 | Lpa200-1 | 24 | Homo | 11.00 | 2 | 5.50 | 7.46 | 1.50 | 4.97 | 13.4 | 1.80 | 57 | 3 | + |
| 38 | Lpa200-2 | 24 | Homo | 11.00 | 2 | 5.50 | 7.46 | 1.50 | 4.97 | 13.5 | 1.81 | 56 | 3 | + |
| Super Bas. (check) | | 84 | Wild Type | 11.00 | 2 | 5.50 | 7.47 | 1.50 | 4.98 | 13.6 | 1.82 | 59 | 3 | + |

^G%: Germination percentage, Homo: Homozygous for low phytate trait, PDL: Paddy length PDW : Paddy Width PDL/PDW : Paddy length width ratio, GL: Grain length, GW: Grain width, GL/GW: Grain length width ratio, CGL: Cooked grain length, ER: Elongation ratio (CGL/GL), HRR: Head rice recovery, B%: Bursting %^

^Aroma: + (present), - (absent)^

Table S3: Colorimetric assay and yield Performance of nine plants in 1^st^ BC_1_F_2:3_populations (Lpa5 × Super Basmati × Super Basmati)

| **Sr .No** | **Progeny** | **PH** | **PT/P** | **PL** | **PB/P** | **F%** | **Lpa test** | **Y/P(gm)** |
| --- | --- | --- | --- | --- | --- | --- | --- | --- |
| 1 | Lpa94 | 129 | 16 | 29.5 | 10 | 92.36 | H | 29.6 |
| 2 | Lpa -106 | 132 | 14 | 30 | 10 | 92.77 | H | 21.6 |
| 3 | Lpa -107 | 124 | 16 | 29 | 11 | 94.94 | H | 23 |
| 4 | Lpa -115 | 134 | 18 | 28 | 10 | 87.79 | H | 23 |
| 5 | Lpa117 | 132 | 17 | 28.5 | 11 | 96.35 | H | 24.2 |
| 6 | Lpa124 | 145 | 18 | 29 | 9 | 88.14 | H | 28.2 |
| 7 | Lpa129 | 146 | 17 | 29.5 | 10 | 72.88 | H | 23.6 |
| 8 | Lpa130 | 145 | 14 | 31.5 | 10 | 91.72 | H | 24.4 |
| 9 | Lpa132 | 144 | 15 | 30 | 9 | 90.48 | H | 27.6 |
| Super basmati (Check) | | 128 | 16 | 29.25 | 12 | 83.00 | N | 21.13 |

_H: heterozygous for low phytate trait, N: negative for low phytate trait_

Table S4: Colorimetric assay and yield Performance of thirty eight plants in 2^nd^ BC_1_F_2:3_populations (Super Basmati × Lpa5 × Super Basmati)

| **Sr .No** | **Progeny** | **PH** | **PT/P** | **PL** | **PB/P** | **F%** | **Lpa test** | **Y/P (gm)** |
| --- | --- | --- | --- | --- | --- | --- | --- | --- |
| 1 | Lpa133 | 129 | 11 | 30.5 | 10 | 94.87 | N | 18.8 |
| 2 | Lpa134 | 134 | 12 | 28.5 | 10 | 91.78 | H | 18.2 |
| 3 | Lpa135 | 137 | 11 | 30.0 | 11 | 94.93 | H | 17.6 |
| 4 | Lpa136 | 128 | 9 | 26.5 | 9 | 93.85 | N | 11.6 |
| 5 | Lpa137 | 124 | 14 | 28.0 | 10 | 91.45 | N | 18.4 |
| 6 | Lpa138 | 128 | 14 | 30.0 | 10 | 94.44 | N | 21.2 |
| 7 | Lpa139 | 132 | 12 | 28.0 | 10 | 95.92 | N | 17.8 |
| 8 | Lpa140 | 140 | 14 | 29.0 | 9 | 91.41 | N | 19.4 |
| 9 | Lpa141 | 132 | 12 | 27.0 | 9 | 91.04 | Homo | 14.4 |
| 10 | Lpa142 | 124 | 15 | 29.0 | 11 | 90.79 | N | 19.8 |
| 11 | Lpa143 | 140 | 13 | 29 | 10 | 94.20 | N | 14.2 |
| 12 | Lpa144 | 132 | 15 | 27.5 | 10 | 89.76 | N | 17.2 |
| 13 | Lpa145 | 128 | 13 | 30 | 9 | 94.44 | N | 20 |
| 14 | Lpa146 | 132 | 15 | 29.5 | 9 | 93.55 | N | 19.2 |
| 15 | Lpa147 | 132 | 14 | 27.5 | 9 | 91.53 | N | 11.8 |
| 16 | Lpa148 | 128 | 14 | 30 | 10 | 95.57 | H | 20.6 |
| 17 | Lpa149 | 127 | 10 | 27 | 9 | 93.38 | N | 12.4 |
| 18 | Lpa150 | 122 | 12 | 19 | 10 | 94.37 | N | 16.2 |
| 19 | Lpa151 | 125 | 10 | 30 | 10 | 96.30 | N | 18.8 |
| 20 | Lpa152 | 127 | 11 | 27 | 9 | 90.32 | N | 16.2 |
| 21 | Lpa153 | 130 | 13 | 28 | 10 | 95.71 | H | 18.2 |
| 22 | Lpa154 | 131 | 13 | 29.5 | 11 | 93.41 | H | 19 |
| 23 | Lpa155 | Data missing | | | | | | |
| 24 | Lpa156 | 128 | 12 | 27 | 11 | 94.93 | N | 17.6 |
| 25 | Lpa157 | 125 | 11 | 29.5 | 10 | 92.86 | N | 15 |
| 26 | Lpa158 | 118 | 15 | 27.5 | 9 | 96.55 | N | 18.8 |
| 27 | Lpa159 | 122 | 11 | 28 | 10 | 94.12 | H | 16.2 |
| 28 | Lpa160 | 131 | 14 | 30 | 10 | 94.35 | N | 17 |
| 29 | Lpa161 | 125 | 16 | 27 | 9 | 90.63 | H | 19.4 |
| 30 | Lpa162 | 125 | 12 | 28 | 11 | 91.55 | N | 17.6 |
| 31 | Lpa163 | 127 | 15 | 29.5 | 10 | 95.81 | N | 23.8 |
| 32 | Lpa164 | 122 | 14 | 28 | 9 | 91.41 | N | 20.4 |
| 33 | Lpa165 | 130 | 15 | 29 | 10 | 92.31 | N | 22.6 |
| 34 | Lpa166 | 129 | 17 | 28.5 | 12 | 94.74 | H | 27.2 |
| 35 | Lpa167 | 137 | 15 | 27.5 | 9 | 90.32 | N | 16.8 |
| 36 | Lpa168 | 119 | 13 | 26.5 | 9 | 94.92 | N | 17 |
| 37 | Lpa169 | 125 | 12 | 29 | 10 | 93.04 | H | 14.4 |
| 38 | Lpa170 | 125 | 17 | 28 | 10 | 94.85 | N | 19.4 |
| **Super basmati** | | 127.67 | 16 | 29.25 | 11.66 | 83 | N | 21.13 |

Table S5: Colorimetric assay and yield Performance of eleven plants in 3rd BC_1_F_2:3_populations (Lpa59 × Super Basmati × Super Basmati)

| **Sr .No** | **Progeny** | **PH** | **PT/P** | **PL** | **PB/P** | **F%** | **Lpa test** | **Y/P (gm)** |
| --- | --- | --- | --- | --- | --- | --- | --- | --- |
| 1 | Lpa70 | 120 | 20 | 30.5 | 11 | 88.89 | H | 28.4 |
| 2 | Lpa71 | 132 | 14 | 31 | 12 | 91.93 | H | 24.8 |
| 3 | Lpa72 | 122 | 14 | 28.5 | 10 | 91.97 | H | 21 |
| 4 | Lpa73 | 131 | 17 | 29.5 | 10 | 94.66 | H | 21.8 |
| 5 | Lpa74 | 131 | 13 | 29 | 11 | 93.38 | H | 19.4 |
| 6 | Lpa75 | 131 | 13 | 29 | 11 | 93.38 | H | 19.4 |
| 7 | Lpa79 | 124 | 21 | 27.5 | 9 | 94.56 | H | 31.2 |
| 8 | Lpa84 | 134 | 14 | 30.5 | 11 | 95.39 | H | 16.6 |
| 9 | Lpa87 | 124 | 15 | 26 | 9 | 91.24 | H | 16.4 |
| 10 | Lpa90 | 123 | 13 | 28 | 9 | 89.92 | H | 16.8 |
| 11 | Lpa91 | 125 | 11 | 25 | 9 | 87.97 | H | 16.0 |
| Super basmati(Check) | | 128 | 16 | 29.25 | 11.66 | 83 | N | 21.1 |

Table S6: Colorimetric assay and yield Performance of fifty three plants in 4th BC_1_F_2:3_ populations (Super Basmati × Lpa59 × Super Basmati)

| **Sr .No** | **Progeny** | **PH** | **PT/P** | **PL** | **PB/P** | **F%** | **Lpa Test** | **Y/P (gm)** |
| --- | --- | --- | --- | --- | --- | --- | --- | --- |
| 1 | Lpa171 | 122 | 13 | 29.0 | 10 | 86.67 | N | 16.8 |
| 2 | Lpa172 | 124 | 13 | 28.5 | 9 | 88.28 | N | 21.4 |
| 3 | Lpa173 | 121 | 10 | 30.0 | 11 | 90.30 | N | 12.0 |
| 4 | Lpa174 | 113 | 10 | 28.5 | 10 | 93.55 | N | 19.2 |
| 5 | Lpa175 | 124 | 12 | 29.5 | 11 | 86.79 | H | 17.4 |
| 6 | Lpa176 | 125 | 11 | 31.5 | 11 | 85.09 | N | 20.8 |
| 7 | Lpa177 | 124 | 13 | 30.5 | 12 | 83.85 | N | 16.8 |
| 8 | Lpa178 | 132 | 13 | 27.5 | 10 | 85.52 | H | 22.4 |
| 9 | Lpa179 | 125 | 8 | 29.5 | 12 | 85.35 | N | 8.40 |
| 10 | Lpa180 | 134 | 13 | 28.0 | 9 | 92.03 | H | 29.4 |
| 12 | Lpa182 | 124 | 11 | 29.0 | 9 | 93.75 | H | 17.8 |
| 13 | Lpa183 | 134 | 13 | 31.0 | 11 | 92.94 | H | 17.2 |
| 14 | Lpa184 | 134 | 14 | 28.0 | 11 | 92.52 | N | 16.6 |
| 15 | Lpa185 | 129 | 12 | 27.5 | 10 | 94.07 | N | 13.6 |
| 16 | Lpa186 | 137 | 16 | 30.5 | 10 | 92.24 | H | 28.6 |
| 17 | Lpa187 | 134 | 11 | 28.0 | 11 | 94.74 | H | 15.4 |
| 18 | Lpa188 | 133 | 17 | 28.0 | 9 | 95.49 | N | 22.6 |
| 19 | Lpa189 | 114 | 13 | 27.0 | 9 | 86.62 | H | 13.4 |
| 20 | Lpa190 | 132 | 17 | 31.0 | 9 | 88.89 | H | 30.8 |
| 21 | Lpa191 | 135 | 14 | 28.0 | 9 | 83.23 | N | 23.4 |
| 22 | Lpa192 | 139 | 13 | 29.5 | 16 | 94.12 | N | 24.6 |
| 23 | Lpa193 | 125 | 13 | 29.0 | 8 | 85.71 | N | 21.6 |
| 24 | Lpa194 | 134 | 12 | 31.0 | 11 | 92.05 | N | 24.8 |
| 25 | Lpa195 | 131 | 14 | 29.0 | 11 | 94.44 | N | 24.8 |
| 26 | Lpa196 | 132 | 12 | 30.0 | 10 | 90.58 | N | 20.4 |
| 27 | Lpa197 | 133 | 14 | 29.0 | 10 | 85.96 | N | 19.2 |
| 28 | Lpa198 | 135 | 14 | 30.5 | 11 | 86.99 | H | 24.8 |
| 29 | Lpa199 | 137 | 17 | 31.0 | 10 | 90.65 | N | 24.6 |
| 30 | Lpa200 | 137 | 9 | 30.0 | 9 | 88.54 | H | 17.6 |
| 31 | Lpa201 | 132 | 11 | 29.0 | 9 | 92.73 | H | 17.8 |
| 32 | Lpa202 | 137 | 15 | 30.0 | 10 | 87.76 | H | 24.4 |
| 33 | Lpa203 | 136 | 12 | 28.0 | 11 | 88.51 | H | 16.4 |
| 34 | Lpa204 | 142 | 14 | 29.5 | 10 | 91.45 | H | 25.4 |
| 35 | Lpa205 | 128 | 12 | 28.0 | 11 | 94.12 | Homo | 15.2 |
| 36 | Lpa206 | 134 | 13 | 28.5 | 10 | 91.55 | N | 20.8 |
| 37 | Lpa207 | 131 | 13 | 29.0 | 10 | 88.16 | H | 19.6 |
| 38 | Lpa208 | 141 | 17 | 31.0 | 11 | 89.78 | N | 31.0 |
| 39 | Lpa209 | 137 | 22 | 38.0 | 11 | 80.33 | H | 28.2 |
| 40 | Lpa210 | 138 | 15 | 27.5 | 10 | 94.67 | N | 17.6 |
| 41 | Lpa211 | 137 | 13 | 30.0 | 11 | 92.50 | N | 21.0 |
| 42 | Lpa212 | 128 | 14 | 28.5 | 9 | 93.24 | H | 21.4 |
| 43 | Lpa213 | 127 | 15 | 29.0 | 9 | 87.18 | H | 15.6 |
| 44 | Lpa214 | 134 | 11 | 28.5 | 11 | 92.25 | N | 24.2 |
| 45 | Lpa215 | 138 | 12 | 29.5 | 10 | 93.48 | H | 20.8 |
| 46 | Lpa216 | 129 | 13 | 29.0 | 10 | 91.77 | N | 13.6 |
| 47 | Lpa217 | 128 | 15 | 28.0 | 10 | 95.80 | N | 16.2 |
| 48 | Lpa218 | 136 | 11 | 29.0 | 11 | 91.18 | H | 20.4 |
| 49 | Lpa219 | 129 | 15 | 29.5 | 10 | 94.37 | N | 20.6 |
| 50 | Lpa220 | 127 | 13 | 29.0 | 10 | 89.76 | N | 17.8 |
| 51 | Lpa221 | 123 | 11 | 28.0 | 9 | 94.31 | H | 15.4 |
| 52 | Lpa222 | 122 | 13 | 27.5 | 11 | 93.66 | N | 18.8 |
| 53 | Lpa223 | 121 | 15 | 28.0 | 10 | 94.52 | H | 16.2 |
| Check (Super Basmati) | | 128 | 16 | 29.25 | 11.66 | 83.00 | N | 21.1 |

^N: negative for low phytate trait H: Heterozygous for low phytate trait, Homo: Pure for low phytate trait^

Table S7: Variation in yield and associated traits among progenies of four back cross populations BC_1_F_3:4_

| Sr. No | Progeny No | PH (cm) | PTP | PL (cm) | PBR | F% | Y/P (gm) | | Lpa Tests |
| --- | --- | --- | --- | --- | --- | --- | --- | --- | --- |
| *Lpa5 × Super Basmati × Super Basmati (*1^st^ BC_1_F_3:4_ population) | | | | | | | | | |
| 1 | Lpa94-1 | 121±3 | 13±4 | 30±1 | 9±1 | 89±2 | 15±4 | | H |
| 2 | Lpa106-1 | 116±9 | 13±4 | 29±2 | 9±1 | 84±6 | 10±2 | | H |
| 3 | Lpa107-1 | 117±8 | 14±3 | 27±1 | 9±1 | 83±4 | 15±3 | | H |
| 4 | Lpa115-1 | 123±3 | 15±3 | 29±1 | 10±1 | 93±1 | 14±2 | | H |
| 5 | Lpa117-1 | 122±7 | 13±2 | 28±5 | 9±1 | 89±2 | 15±1 | | H |
| 6 | Lpa124-1 | 119±9 | 10±2 | 27±1 | 9±1 | 90±2 | 13±3 | | N |
| 7 | Lpa129-1 | 122±7 | 13±4 | 28±3 | 10±1 | 93±3 | 14±5 | | N |
| 8 | Lpa130-1 | 121±7 | 12±1 | 28±2 | 10±2 | 89±3 | 13±3 | | H |
| 9 | Lpa132-1 | 120±3 | 13±1 | 29±1 | 10±1 | 93±3 | 16±2 | | H |
| *Super Basmati × Lpa5 × Super Basmati (2^nd^ BC_1_F_3:4_ Population)* | | | | | | | | | |
| 1 | Lpa133-1 | 114±7 | 13±1 | 28±1 | 10±1 | 87±2 | 12±2 | | N |
| 2 | Lpa134-1 | 116±3 | 13±3 | 27±1 | 9±1 | 86±2 | 12±2 | | H |
| 3 | Lpa135-1 | 115±4 | 11±2 | 27±2 | 10±2 | 88±3 | 10±1 | | N |
| 4 | Lpa136-1 | 112±3 | 10±3 | 26±1 | 9±1 | 88±2 | 9±4 | | N |
| 5 | Lpa137-1 | 113±3 | 10±3 | 27±1 | 9±1 | 85±2 | 10±4 | | N |
| 6 | Lpa138-1 | 125±4 | 12±5 | 27±2 | 9±1 | 83±6 | 11±5 | | N |
| 7 | Lpa139-1 | 117±4 | 13±3 | 28±2 | 9±1 | 87±3 | 12±4 | | N |
| 8 | Lpa140-1 | 125±1 | 7±5 | 28±3 | 8±2 | 84±4 | 9±6 | | N |
| 9 | Lpa141-4 | 109±2 | 10±2 | 27±2 | 10±1 | 88±1 | 14±1 | | Homo |
| 10 | Lpa142-1 | 108±8 | 10±1 | 25±2 | 9±1 | 91±3 | 8±2 | | N |
| 11 | Lpa143-1 | 122±9 | 15±4 | 28±2 | 9±1 | 87±4 | 11±3 | | N |
| 12 | Lpa144-1 | 116±1 | 10±2 | 27±2 | 9±1 | 90±3 | 8±2 | | N |
| 13 | Lpa145-1 | 111±9 | 11±2 | 27±2 | 10±1 | 89±3 | 9±4 | | N |
| 14 | Lpa146-1 | 113±4 | 11±3 | 27±1 | 9±1 | 87±5 | 10±3 | | N |
| 15 | Lpa147-1 | 114±10 | 11±2 | 25±3 | 9±1 | 89±5 | 11±2 | | N |
| 16 | Lpa148-1 | 111±2 | 13±5 | 29±1 | 10±1 | 88±4 | 14±3 | | H |
| 17 | Lpa149-1 | 111±3 | 8±2 | 27±1 | 10±2 | 83±10 | 8±1 | | N |
| 18 | Lpa150-1 | 120±5 | 12±1 | 27±1 | 11±2 | 88±10 | 11±2 | | N |
| 19 | Lpa151-1 | 121±5 | 11±1 | 28±1 | 10±1 | 88±6 | 11±2 | | N |
| 20 | Lpa152-1 | 121±2 | 11±1 | 27±1 | 10±1 | 84±5 | 10±2 | | N |
| 21 | Lpa153-1 | 126±6 | 11±1 | 26±1 | 10±1 | 90±6 | 12±2 | | H |
| 22 | Lpa154-1 | 125±4 | 13±3 | 27±1 | 10±1 | 89±3 | 14±4 | | H |
| 23 | Lpa156-1 | 122±5 | 10±2 | 26±1 | 10±1 | 86±4 | 9±2 | | N |
| 24 | Lpa157-1 | 118±3 | 12±2 | 28±2 | 9±1 | 91±1 | 10±1 | | N |
| 25 | Lpa158-1 | 119±5 | 10±1 | 27±3 | 9±1 | 89±3 | 13±2 | | N |
| 26 | Lpa159-1 | 121±5 | 13±3 | 28±1 | 9±1 | 88±3 | 17±4 | | H |
| 27 | Lpa160-1 | 121±6 | 12±2 | 30±1 | 10±1 | 87±2 | 13±2 | | N |
| 28 | Lpa161-1 | 130±4 | 15±3 | 26±1 | 9±0 | 91±1 | 18±3 | | H |
| 29 | Lpa162-1 | 116±4 | 10±2 | 25±2 | 9±1 | 87±3 | 11±3 | | N |
| 30 | Lpa163-1 | 114±4 | 10±2 | 29±2 | 10±1 | 89±6 | 12±5 | | N |
| 31 | Lpa164-1 | 118±6 | 10±4 | 28±1 | 10±1 | 89±5 | 12±3 | | N |
| 32 | Lpa165-1 | 119±3 | 11±1 | 26±1 | 10±0 | 92±2 | 11±2 | | N |
| 33 | Lpa166-1 | 112±5 | 10±3 | 27±2 | 10±1 | 93±2 | 10±5 | | H |
| 34 | Lpa167-1 | 124±6 | 10±1 | 28±1 | 10±1 | 90±3 | 11±3 | | N |
| 35 | Lpa168-1 | 110±5 | 7±2 | 25±1 | 9±1 | 92±4 | 8±1 | | N |
| 36 | Lpa169-1 | 115±3 | 10±1 | 26±2 | 10±1 | 90±4 | 11±2 | | H |
| 37 | Lpa170-1 | 123±4 | 11±4 | 27±2 | 9±1 | 90±4 | 10±2 | | N |
| *Lpa59 × Super Basmati × Super Basmati (3^rd^ BC_1_F_3:4_ population)* | | | | | | | | | |
| 1 | Lpa70-1 | 117±8 | 13±3 | 29±2 | 10±1 | 91±2 | 17±3 | H | |
| 2 | Lpa71-1 | 122±6 | 14±2 | 28±1 | 10±1 | 91±3 | 18±1 | H | |
| 3 | Lpa72-1 | 122±7 | 13±1 | 29±1 | 10±1 | 90±1 | 15±2 | H | |
| 4 | Lpa73-1 | 121±5 | 17±3 | 28±1 | 10±1 | 91±3 | 16±3 | H | |
| 5 | Lpa74-1 | 120±6 | 16±3 | 29±3 | 10±1 | 89±2 | 14±3 | H | |
| 6 | Lpa75-1 | 118±5 | 14±4 | 28±2 | 9±1 | 92±4 | 14±4 | H | |
| 7 | Lpa79-1 | 120±5 | 13±1 | 30±2 | 11±1 | 90±3 | 16±3 | H | |
| 8 | Lpa84-1 | 120±7 | 13±1 | 28±3 | 10±1 | 88±4 | 14±3 | H | |
| 9 | Lpa87-1 | 123±8 | 14±2 | 30±2 | 10±1 | 88±4 | 17±2 | H | |
| 10 | Lpa90-1 | 114±4 | 13±1 | 29±1 | 10±1 | 93±4 | 14±2 | H | |
| 11 | Lpa91-1 | 119±3 | 15±3 | 29±1 | 10±1 | 88±5 | 16±3 | H | |
| *Super Basmati × Lpa59× Super Basmati (4^th^BC_1_F_3:4_ population)* | | | | | | | | | |
| 1 | Lpa171-4 | 117±4 | 14±3 | 30±1 | 10±1 | 87±4 | 16±2 | N | |
| 2 | Lpa172-1 | 121±3 | 16±2 | 29±1 | 10±1 | 89±3 | 15±1 | N | |
| 3 | Lpa173-1 | 127±3 | 12±1 | 29±2 | 10±1 | 92±3 | 14±3 | N | |
| 4 | Lpa174-1 | 122±4 | 15±1 | 30±1 | 11±1 | 87±4 | 17±2 | N | |
| 5 | Lpa175-1 | 130±4 | 16±3 | 29±1 | 11±1 | 92±2 | 19±4 | H | |
| 6 | Lpa176-1 | 123±5 | 17±1 | 28±2 | 9±1 | 86±3 | 15±3 | N | |
| 7 | Lpa177-1 | 121±2 | 17±2 | 27±2 | 10±1 | 86±3 | 15±1 | N | |
| 8 | Lpa178-1 | 123±4 | 13±4 | 25±1 | 9±1 | 86±3 | 13±4 | H | |
| 9 | Lpa179-1 | 126±4 | 13±1 | 30±2 | 10±1 | 88±3 | 15±3 | N | |
| 10 | Lpa180-1 | 131±2 | 15±1 | 28±2 | 10±1 | 84±3 | 18±1 | H | |
| 11 | Lpa181-1 | 119±5 | 16±3 | 28±2 | 10±1 | 87±2 | 15±3 | H | |
| 12 | Lpa182-1 | 122±6 | 15±3 | 28±1 | 10±1 | 85±6 | 14±4 | H | |
| 13 | Lpa184-1 | 128±4 | 19±6 | 28±2 | 11±1 | 85±5 | 18±5 | N | |
| 14 | Lpa185-1 | 122±3 | 14±2 | 27±3 | 10±1 | 88±1 | 16±6 | N | |
| 15 | Lpa186-1 | 117±3 | 14±3 | 27±3 | 9±1 | 89±2 | 13±4 | H | |
| 16 | Lpa187-1 | 126±3 | 15±3 | 28±3 | 10±1 | 83±3 | 16±1 | H | |
| 17 | Lpa188-1 | 116±1 | 16±3 | 29±1 | 10±1 | 85±1 | 15±2 | N | |
| 18 | Lpa189-1 | 119±4 | 16±2 | 26±1 | 10±1 | 86±1 | 17±5 | H | |
| 19 | Lpa190-1 | 125±6 | 17±2 | 28±2 | 10±1 | 86±2 | 19±4 | H | |
| 20 | Lpa191-1 | 120±7 | 16±1 | 30±2 | 10±1 | 84±1 | 19±2 | N | |
| 21 | Lpa192-1 | 126±3 | 13±2 | 29±2 | 10±1 | 77±3 | 14±2 | N | |
| 22 | Lpa193-1 | 122±3 | 16±1 | 29±2 | 10±1 | 82±2 | 15±2 | N | |
| 23 | Lpa194-1 | 131±4 | 14±5 | 29±3 | 10±2 | 87±5 | 17±3 | N | |
| 24 | Lpa195-1 | 110±2 | 12±1 | 28±2 | 10±1 | 86±4 | 10±2 | N | |
| 25 | Lpa196-1 | 124±7 | 13±2 | 27±1 | 11±2 | 86±2 | 11±6 | N | |
| 26 | Lpa197-1 | 118±4 | 15±3 | 26±2 | 10±1 | 86±4 | 12±4 | N | |
| 27 | Lpa198-1 | 125±5 | 11±3 | 29±2 | 10±1 | 88±3 | 12±3 | H | |
| 28 | Lpa199-1 | 120±5 | 15±3 | 29±1 | 11±1 | 89±3 | 14±5 | N | |
| 29 | Lpa200-1 | 124±4 | 13±2 | 28±2 | 11±1 | 87±5 | 11±4 | H | |
| 30 | Lpa201-1 | 102±1 | 12±4 | 23±1 | 9±1 | 84±9 | 16±2 | H | |
| 31 | Lpa202-1 | 120±3 | 11±2 | 29±1 | 10±1 | 91±2 | 15±2 | H | |
| 32 | Lpa203-1 | 124±5 | 15±2 | 29±2 | 9±1 | 89±3 | 17±1 | H | |
| 33 | Lpa204-1 | 116±3 | 10±1 | 24±1 | 9±1 | 89±3 | 11±4 | H | |
| 34 | Lpa205-4 | 107±9 | 13±4 | 29±1 | 10±2 | 86±5 | 12±7 | Homo | |
| 35 | Lpa206-1 | 124±5 | 16±3 | 28±1 | 10±1 | 86±4 | 17±1 | N | |
| 36 | Lpa207-1 | 124±5 | 18±3 | 29±2 | 9±1 | 88±2 | 18±2 | H | |
| 37 | Lpa208-1 | 119±8 | 17±4 | 28±2 | 10±1 | 88±1 | 18±8 | N | |
| 38 | Lpa209-1 | 121±5 | 13±2 | 29±2 | 10±1 | 90±2 | 16±2 | H | |
| 39 | Lpa210-1 | 119±3 | 16±4 | 29±1 | 10±1 | 86±6 | 15±2 | N | |
| 40 | Lpa211-1 | 122±6 | 15±4 | 27±1 | 11±1 | 88±3 | 14±5 | N | |
| 41 | Lpa212-1 | 113±1 | 15±2 | 28±1 | 9±0 | 93±3 | 15±3 | H | |
| 42 | Lpa213-1 | 110±3 | 19±3 | 27±1 | 9±1 | 85±4 | 20±3 | H | |
| 43 | Lpa214-1 | 118±1 | 15±3 | 27±1 | 9±1 | 91±4 | 16±6 | N | |
| 44 | Lpa215-1 | 122±5 | 15±3 | 24±1 | 9±1 | 90±4 | 17±2 | H | |
| 45 | Lpa216-1 | 116±1 | 15±2 | 27±3 | 9±1 | 87±4 | 16±1 | N | |
| 46 | Lpa217-1 | 118±4 | 10±1 | 28±1 | 9±1 | 87±2 | 11±3 | N | |
| 47 | Lpa218-1 | 123±5 | 12±2 | 26±2 | 10±1 | 89±2 | 14±3 | H | |
| 48 | Lpa219-1 | 114±3 | 14±5 | 28±1 | 11±1 | 89±5 | 17±7 | N | |
| 49 | Lpa220-1 | 118±2 | 10±2 | 25±1 | 10±1 | 88±3 | 11±3 | N | |
| 50 | Lpa221-1 | 121±3 | 16±2 | 28±1 | 10±1 | 92±1 | 15±2 | H | |
| 51 | Lpa222-1 | 122±6 | 10±2 | 27±2 | 9±1 | 86±4 | 11±3 | N | |
| 52 | Lpa223-1 | 107±9 | 13±5 | 28±2 | 10±1 | 89±4 | 13±6 | H | |

_H: Heterozygous for low phytate trait N: Negative for low phytate trait Homo: Homozygous for low phytate trait_

|  |
| --- |
|  |


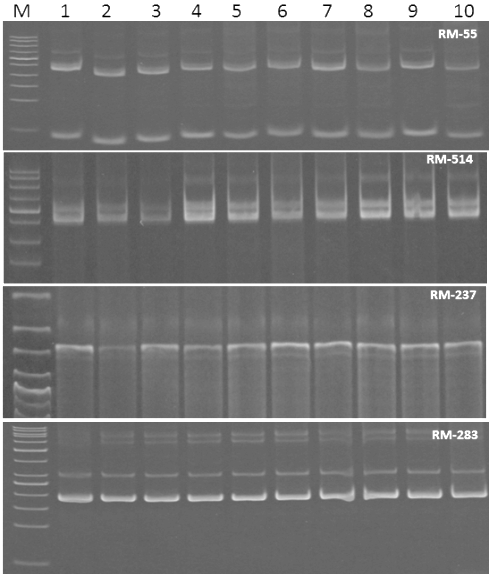


Figure S1: Electrophorogram showing microsatellite profiles of the parental genotype and low phytate mutants using different primers resolved on 6% PAGE.Lane: 1-9, Lpa5, Lpa6, Lpa7, Lpa30, Lpa88, Lpa153, Lpa239, Lpa244 and Lpa257 respectively, Lane: 10, Super basmati, M: DNA ladder iVDye 50bp DNA ladder (GenDEPOT)


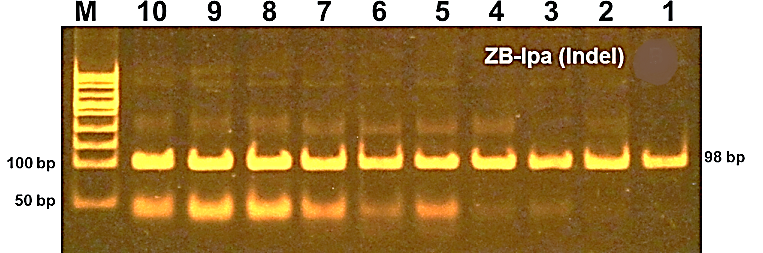


Figure S2: Electrophorogram showing ZB-Lpa (indel) profiles of the parental genotype and low phytate mutants using different primers. Lane: 1-9, Lpa5, Lpa6, Lpa7, Lpa30, Lpa88, Lpa153, Lpa239, Lpa244 and Lpa257 respectively, Lane: 10, Super basmati, M: 50bp DNA ladder (GM305 Biobasic).


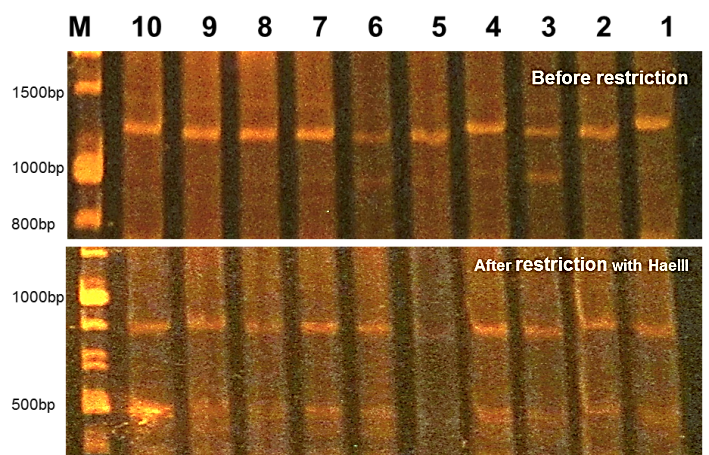


Figure S3: Electrophorogram showing profiles of the parental genotype and low phytate mutants using XS-Lpa primers resolved on 1% Agarose gel. Lane: 1-9, Lpa5, Lpa6, Lpa7, Lpa30, Lpa88, Lpa153, Lpa239, Lpa244 and Lpa257 respectively, Lane: 10, Super basmati, M: DNA 1 kb (+) DNA Ladder Marker (Cat.# DM003 Enzynomics).
